# Building a 5-HT3A Receptor Expression Map in the Mouse Brain

**DOI:** 10.1038/srep42884

**Published:** 2017-03-09

**Authors:** Yoshihisa Koyama, Makoto Kondo, Shoichi Shimada

**Affiliations:** 1 Department of Neuroscience and Cell Biology, Osaka University Graduate School of Medicine, Osaka 565-0871, Japan

## Abstract

Of the many serotonin receptors, the type 3 receptors (5-HT3R) are the only ionotropic ones, playing a key role in fast synaptic transmission and cognitive and emotional brain function through controlled neuronal excitation. To better understand the various functions of 5-HT3Rs, it is very important to know their expression pattern in the central nervous system (CNS). To date, many distributional studies have shown localized 5-HT3R expression in the brain and spinal cord. However, an accurate pattern of 5-HT3R expression in the CNS remains to be elucidated. To investigate the distribution of 5-HT3R in the mouse brain in detail, we performed immunofluorescent staining using 5-HT3AR-GFP transgenic mice. We found strong 5-HT3AR expression in the olfactory bulb, cerebral cortex, hippocampus, and amygdala; and partial expression in the pons, medulla, and spinal cord. Meanwhile, the thalamus, hypothalamus, and midbrain exhibited a few 5-HT3AR-expressing cells, and no expression was detected in the cerebellum. Further, double-immunostaining using neural markers confirmed that 5-HT3AR is expressed in GABAergic interneurons containing somatostatin or calretinin. In the present study, we built a 5-HT3AR expression map in the mouse brain. Our findings make significant contributions in elucidating the novel functions of 5-HT3R in the CNS.

Serotonin (5-hydroxytryptamine, 5-HT) is a common neurotransmitter in mammals, and the serotonergic system plays an essential role in the regulation of various behaviours such as sleep, perception, and cognitive and autonomic functions in the mammalian central nervous system (CNS). Recent cloning techniques and pharmacological analyses have identified 14 distinct 5-HT receptor (5-HTR) subtypes classified into seven receptor families (from 5-HT1R to 5-HT7R) on the basis of their structural, functional, and pharmacological properties^1^. Unlike all the other 5-HTRs, which are G-protein-coupled receptors, 5-HT3R belongs to the Cys-loop receptor family of pentameric neurotransmitter-gated ion channels and is involved in fast serotonin neurotransmission^2^. The 5-HT3AR subunit is essential for the formation of a functional receptor^3^.

Previous studies have investigated the distribution pattern of 5-HT3R in the brain. Autoradiographic analyses have mainly been performed using the following selective drugs for labelling 5-HT3R in the mammalian brain: [3H]-metachlorophenylbiguanide^4^,^5^; [3H]-quipazine^6^,^7^; [3H]-tropisetron^8^,^9^; [3H]-zacopride, [3H]-(S)-zacopride, [125I]-zacopride^10^,^11^,^12^,^13^,^14^,^15^,^16^; [3H]-granisetron^17^,^18^; [3H]-LY278584^19^; and [3H]-GR65630^20^,^21^,^22^,^23^,^24^,^25^. In addition, *in situ* hybridization^26^,^27^ and immunohistochemical studies using antibodies specific to 5-HT3R^28^,^29^,^30^,^31^,^32^,^33^ have been performed in various rodent species. These findings have shown that the highest level of expression of 5-HT3R can be observed in the spinal cord (especially the superficial layer of the dorsal horn), spinal trigeminal nucleus (Sp5), and the dorsal medulla oblongata containing the area postrema (AP), the nucleus of the solitary tract (NTS), and the dorsal motor nucleus of the vagus nerve (DMV). Moreover, strong 5-HT3R signals have also been detected in specific regions of the cerebral cortex including the hippocampal formation and in some subnuclei of the amygdaloid body. The expression level in the basal ganglia and midbrain structures is low.

Previous studies have indicated that 5-HT3R is involved in cognitive and emotional brain processes such as spatial memory^34^, fear extinction^35^, anxiety-like behaviour^36^, and exercise-induced antidepressant effects^37^. The implication that 5-HT3R plays a role in these behaviours is consistent with previous findings of its high expression in limbic regions such as the hippocampus, amygdala, and prefrontal cortex^26^,^38^,^39^. However, the current knowledge of 5-HT3R expression patterns in the CNS is somewhat limited and insufficient for understanding the functional diversity of 5-HT3Rs. Therefore, in the present study, we used commercial transgenic mice expressing enhanced green fluorescent protein (GFP) under the control of the 5-HT3AR promoter (5-HT3AR-GFP TG mice^40^,^41^) and performed immunohistochemical analysis to examine highly detailed expression profiles of 5-HT3R in the CNS. Our findings suggest more extensive functions of the 5-HT3R and possible therapeutic targets for various CNS disorders.

## Results

### Validation of GFP expression in 5-HT3AR-GFP TG mice

**Figure 1:**
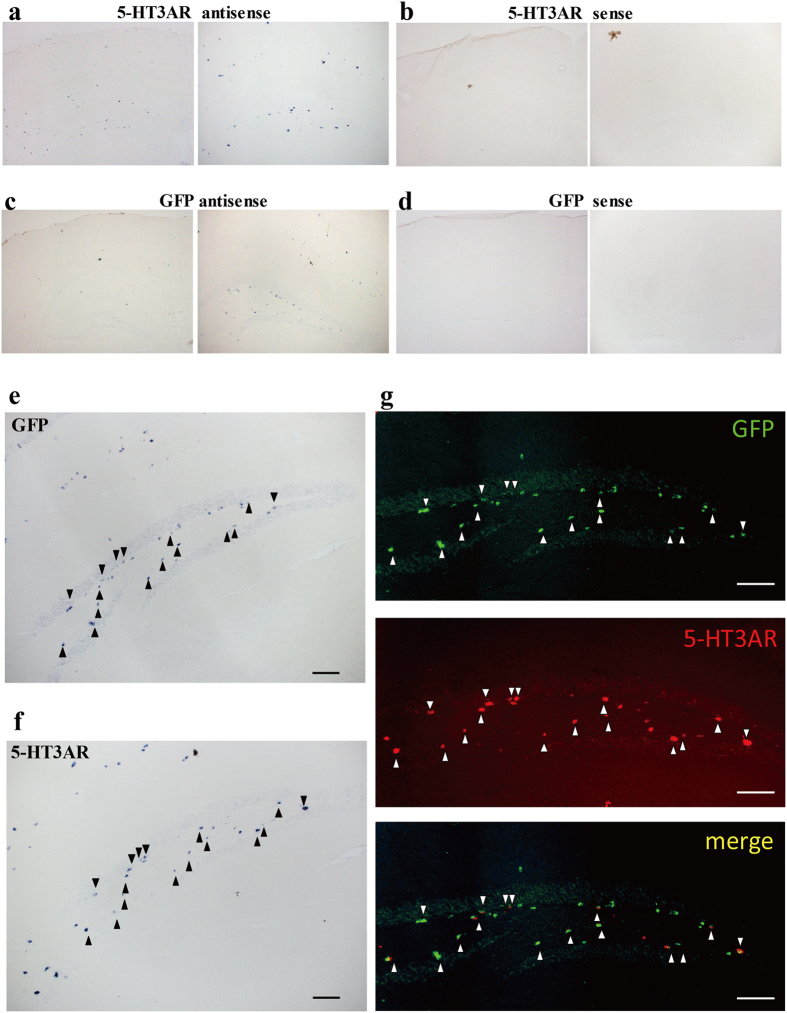
Validation of GFP expression in 5-HT3AR-GFP-TG mice. (**a–d**) Micrograph of *in situ* hybridization for 5-HT3AR mRNA (**a**: antisense; **b**: sense) and GFP mRNA (**c**: antisense; **d**: sense) in coronal 5-HT3AR-GFP TG mouse sections containing the hippocampal formation, at low (left panel) and high magnification (right panel). Scale bar: 400 μm (left) and 200 μm (right). (**e–i**) *In situ* hybridization analysis using mirror-image sections; micrographs for GFP mRNA (**e**) and 5-HT3AR mRNA (**f**); arrowheads indicate positively labelled cells. Scale bar: 50 μm. (**g**) False colour images of *in situ* hybridization for GFP mRNA (upper: green), 5-HT3AR mRNA (middle: red), and merged (bottom); arrowheads indicate positively labelled cells. Scale bar: 50 μm.

To assess whether GFP-positive cells in the 5-HT3AR-GFP TG mice truly expressed 5-HT3AR, we performed *in situ* hybridization with DIG-labelled probes using coronal brain sections containing the hippocampus. Initially, serial sections were hybridized with both antisense and sense probes to confirm the specificity of the hybridized signals for both GFP and 5-HT3AR mRNA. The expression of both mRNAs was detected in the hippocampus and was strong in the DG, while no signal was observed in consecutive sections hybridized with either of the sense probes (Fig. 1a–d). Subsequently, to examine the co-expression of GFP and 5-HT3AR, *in situ* hybridization using mirror image sections of the DG was performed. Both the mRNAs were expressed in the subgranular zone, partially in the dentate hilus, and not in the granular layer (Fig. 1e,f). Moreover, false colour merged images demonstrated that GFP and 5-HT3AR were largely expressed in the same cells in the subgranular zone, but scarcely in the dentate hilus (Fig. 1g, arrowhead). Therefore, these results suggest that almost all the GFP-positive cells in 5-HT3AR-GFP TG mice actually expressed 5-HT3AR.

### Distribution of 5-HT3AR-expressing cells in the CNS

**Figure 2:**
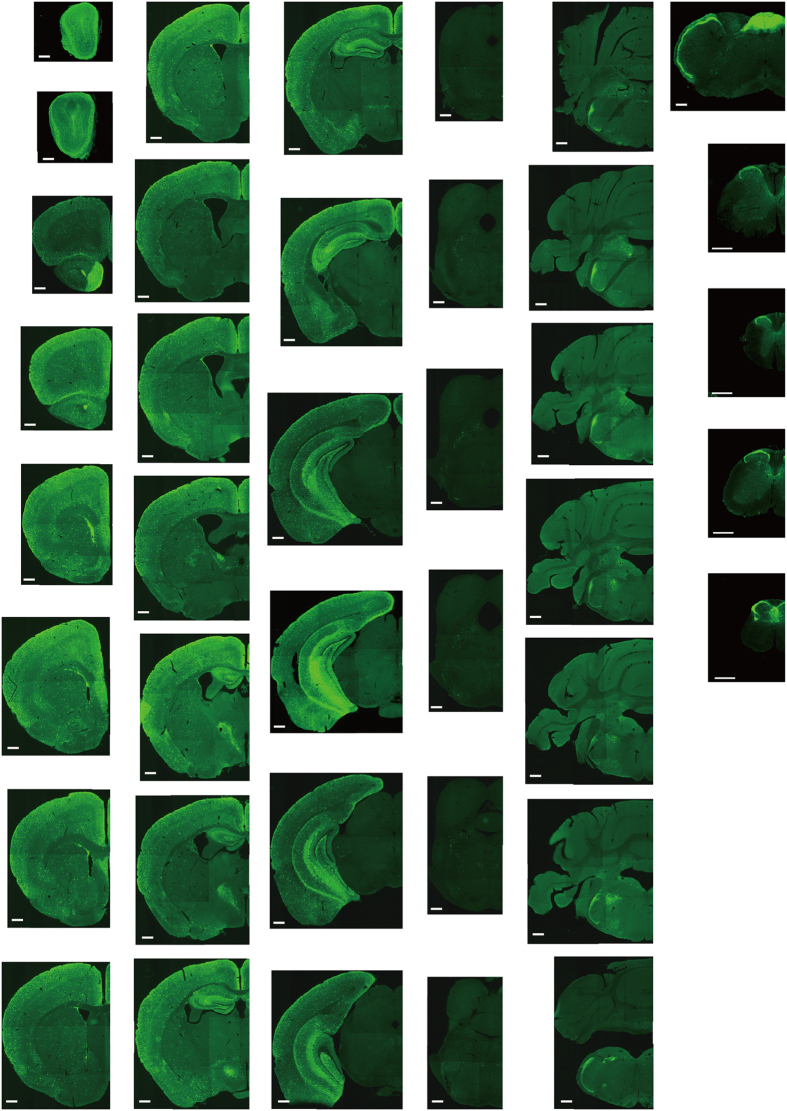
Overview of 5-HT3AR-expressing cells in the CNS. The series of GFP immunofluorescent staining images of coronal sections from the olfactory bulb to the sacral cord of 5-HT3AR-TG mice. Scale bar: 500 μm.

**Figure 3:**
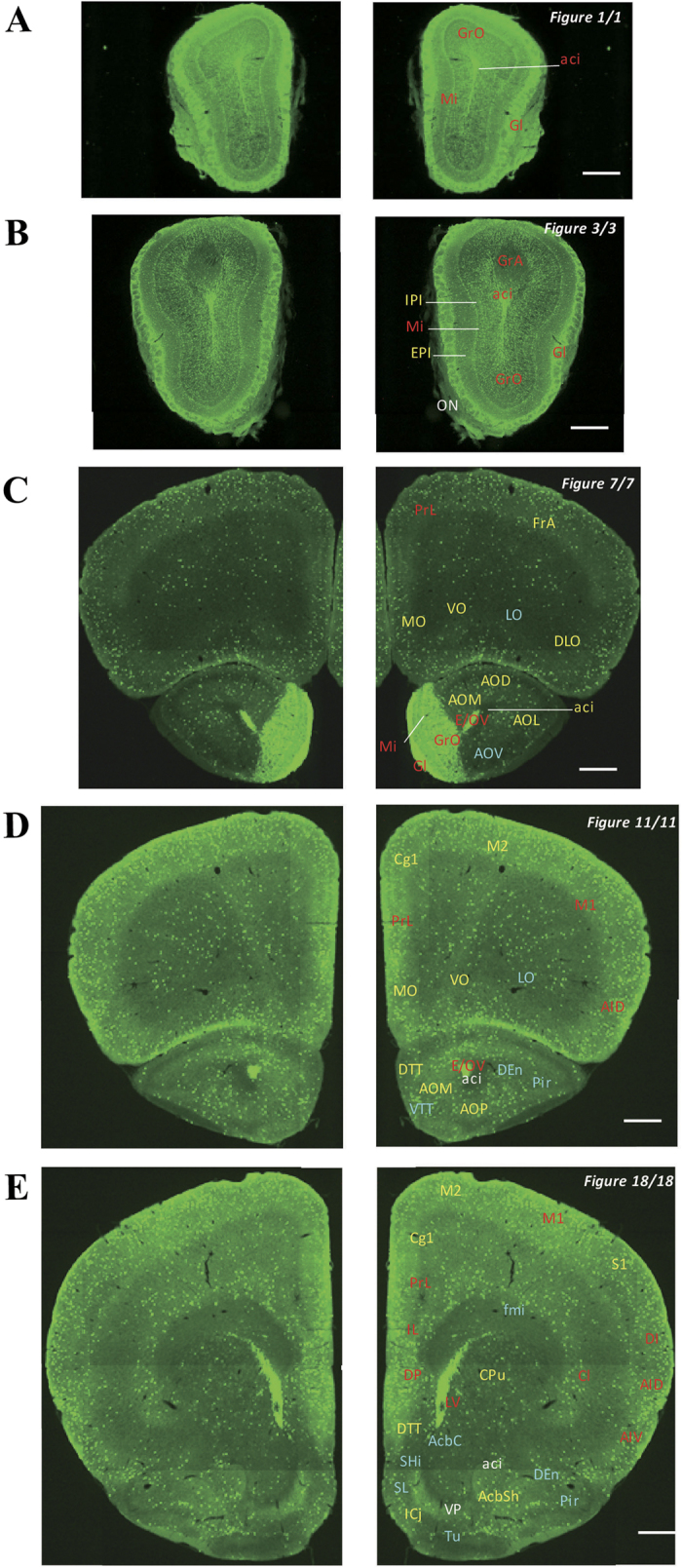
Detailed distribution of 5-HT3AR-expressing cells in the olfactory bulb and forebrain. The series of GFP immunofluorescent staining images of coronal sections of 5-HT3AR-TG mice from 1 to 18 (after-mentioned atras figure); Fluorescence images (left) and the abbreviation-tagged images (right); each abbreviation is linked to Supplementary Table 1 and is coloured depending on the expression intensity: strong = red; moderately high = yellow; weak = blue; negative = white. The figure number in the upper right refers to two brain atlases (the Mouse Brain in Stereotaxic Coordinates 2nd edition and the Atlas of the Spinal Cord: Mouse, Rat, Rhesus, Marmoset, and Human). Scale bar: 500 µm.

**Figure 4:**
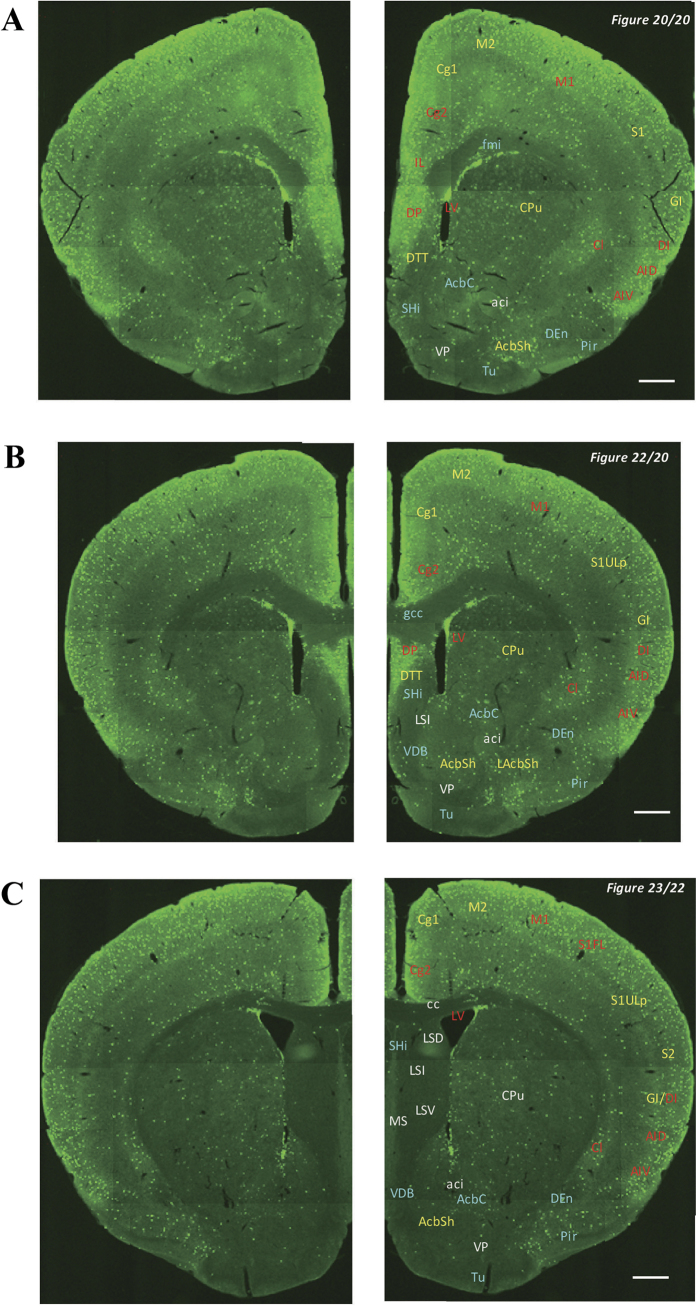
Detailed distribution of 5-HT3AR-expressing cells in the striatum, nucleus accumbens and forebrain. The series of GFP immunofluorescent staining images of coronal sections of 5-HT3AR-TG mice from 20 to 23 (after-mentioned atras figure); Fluorescence images (left) and the abbreviation-tagged images (right); each abbreviation is linked to Table 1 and is coloured depending on the expression intensity: strong = red; moderately high = yellow; weak = blue; negative = white. The figure number in the upper right refers to two brain atlases (the Mouse Brain in Stereotaxic Coordinates 2nd edition and the Atlas of the Spinal Cord: Mouse, Rat, Rhesus, Marmoset, and Human). Scale bar: 500 µm.

**Figure 5:**
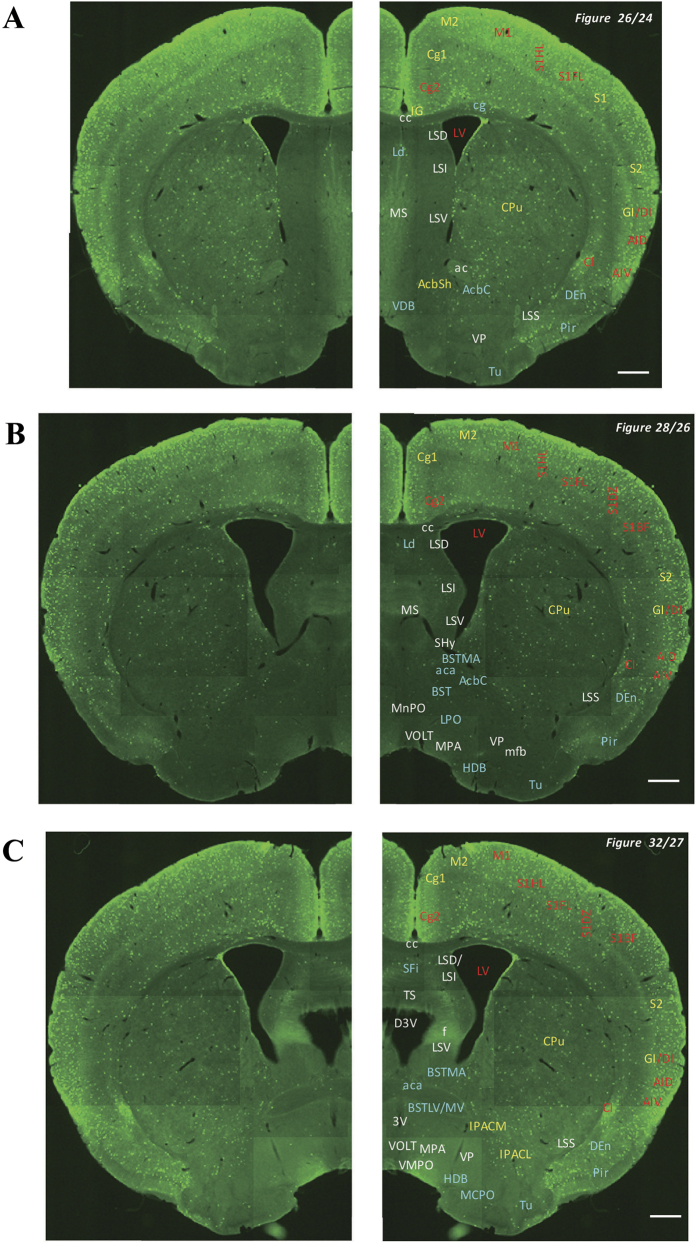
Detailed distribution of 5-HT3AR-expressing cells in the striatum, cerebral cortex and septal region. The series of GFP immunofluorescent staining images of coronal sections of 5-HT3AR-TG mice from 24 to 32 (after-mentioned atras figure); Fluorescence images (left) and the abbreviation-tagged images (right); each abbreviation is linked to Table 1 and is coloured depending on the expression intensity: strong = red; moderately high = yellow; weak = blue; negative = white. The figure number in the upper right refers to two brain atlases (the Mouse Brain in Stereotaxic Coordinates 2nd edition and the Atlas of the Spinal Cord: Mouse, Rat, Rhesus, Marmoset, and Human). Scale bar: 500 µm.

**Figure 6:**
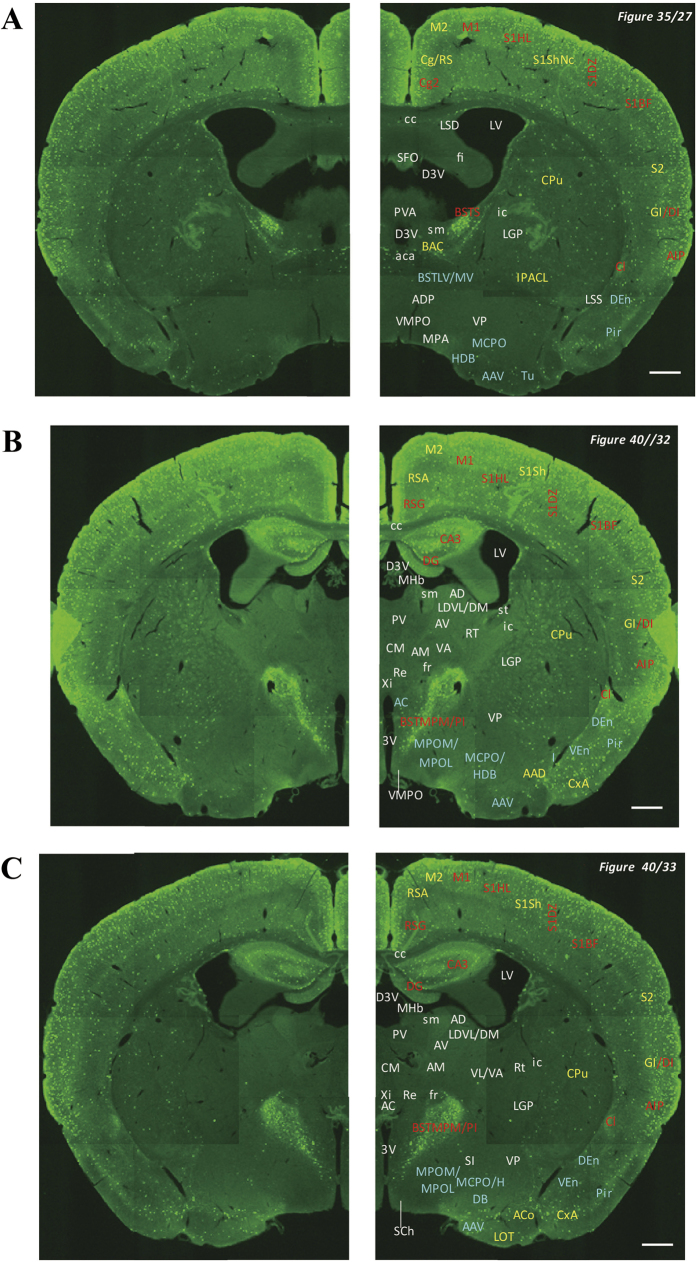
Detailed distribution of 5-HT3AR-expressing cells in the bed nucleus of stria terminalis cerebral cortex and diencephalon. The series of GFP immunofluorescent staining images of coronal sections of 5-HT3AR-TG mice from 27 to 40 (after-mentioned atras figure); Fluorescence images (left) and the abbreviation-tagged images (right); each abbreviation is linked to Table 1 and is coloured depending on the expression intensity: strong = red; moderately high = yellow; weak = blue; negative = white. The figure number in the upper right refers to two brain atlases (the Mouse Brain in Stereotaxic Coordinates 2nd edition and the Atlas of the Spinal Cord: Mouse, Rat, Rhesus, Marmoset, and Human). Scale bar: 500 µm.

**Figure 7:**
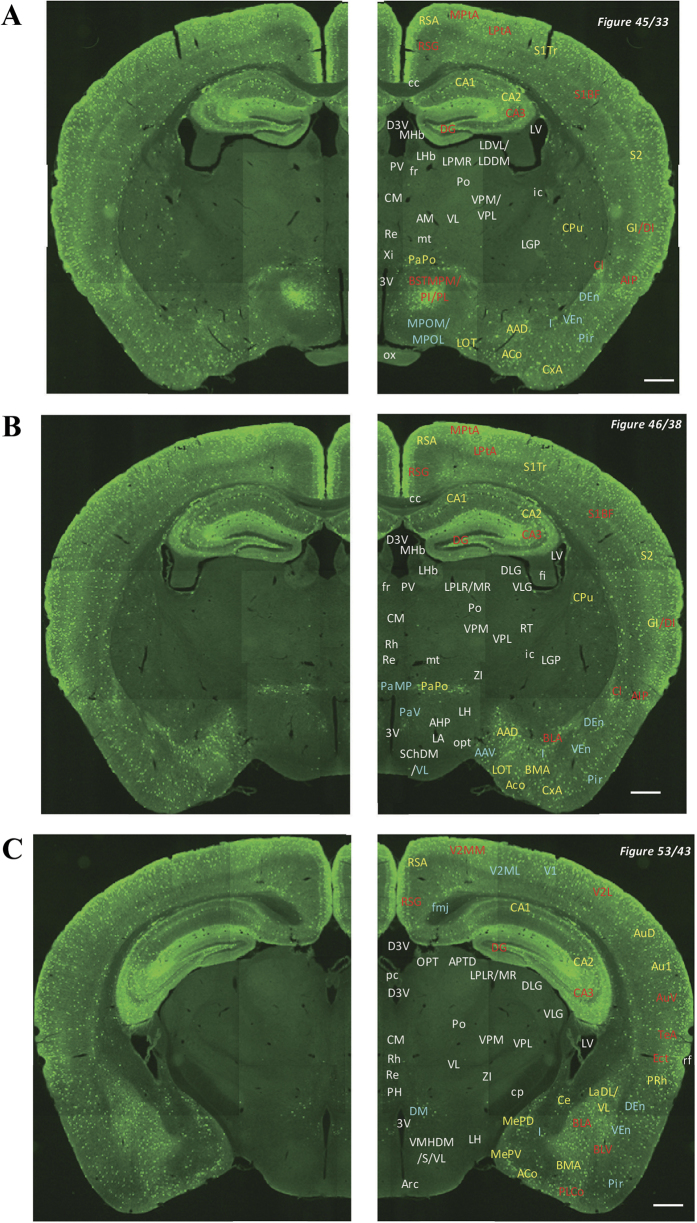
Detailed distribution of 5-HT3AR-expressing cells in the cerebral cortex, hippocampus, amygdala and diencephalon. The series of GFP immunofluorescent staining images of coronal sections of 5-HT3AR-TG mice from 33 to 53 (after-mentioned atras figure); Fluorescence images (left) and the abbreviation-tagged images (right); each abbreviation is linked to Table 1 and is coloured depending on the expression intensity: strong = red; moderately high = yellow; weak = blue; negative = white. The figure number in the upper right refers to two brain atlases (the Mouse Brain in Stereotaxic Coordinates 2nd edition and the Atlas of the Spinal Cord: Mouse, Rat, Rhesus, Marmoset, and Human). Scale bar: 500 µm.

**Figure 8:**
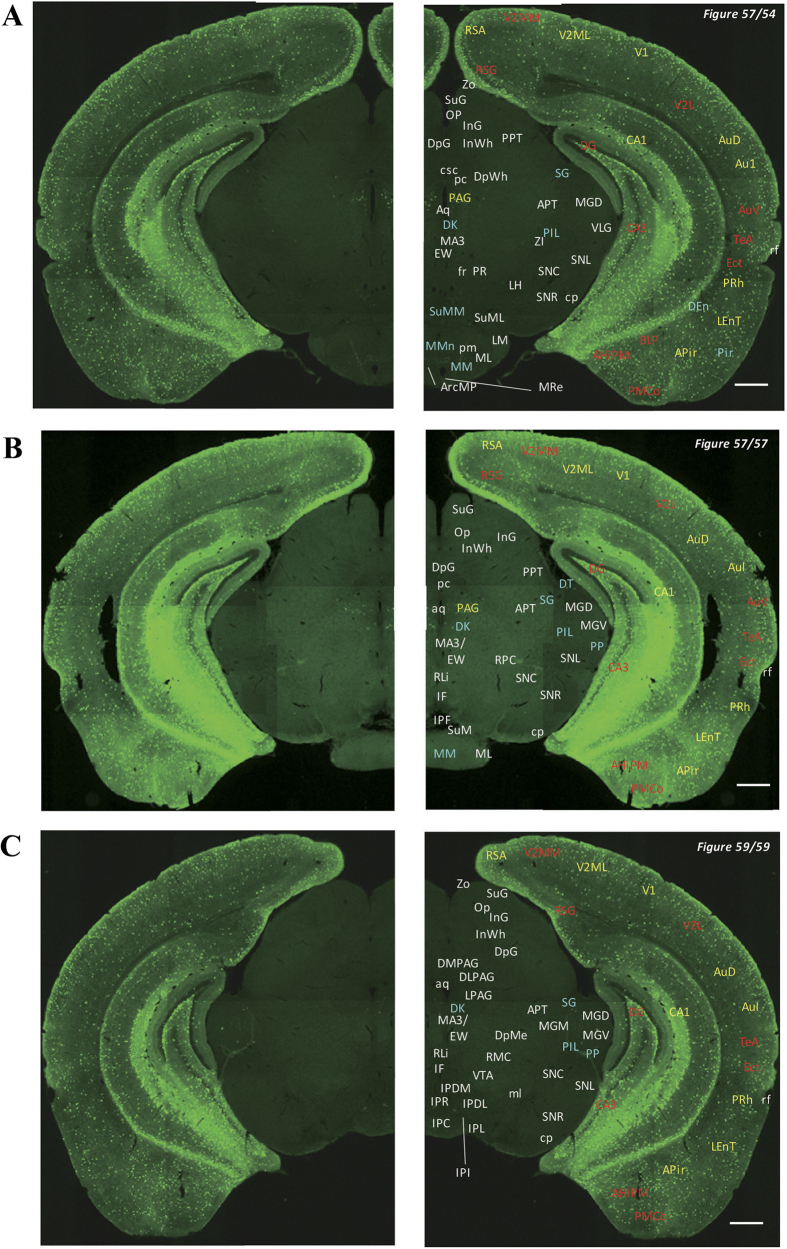
Detailed distribution of 5-HT3AR-expressing cells in the rostral midbrain, mammillary body and adjacent cerebral cortex. The series of GFP immunofluorescent staining images of coronal sections of 5-HT3AR-TG mice from 54 to 59 (after-mentioned atras figure); Fluorescence images (left) and the abbreviation-tagged images (right); each abbreviation is linked to Table 1 and is coloured depending on the expression intensity: strong = red; moderately high = yellow; weak = blue; negative = white. The figure number in the upper right refers to two brain atlases (the Mouse Brain in Stereotaxic Coordinates 2nd edition and the Atlas of the Spinal Cord: Mouse, Rat, Rhesus, Marmoset, and Human). Scale bar: 500 µm.

**Figure 9:**
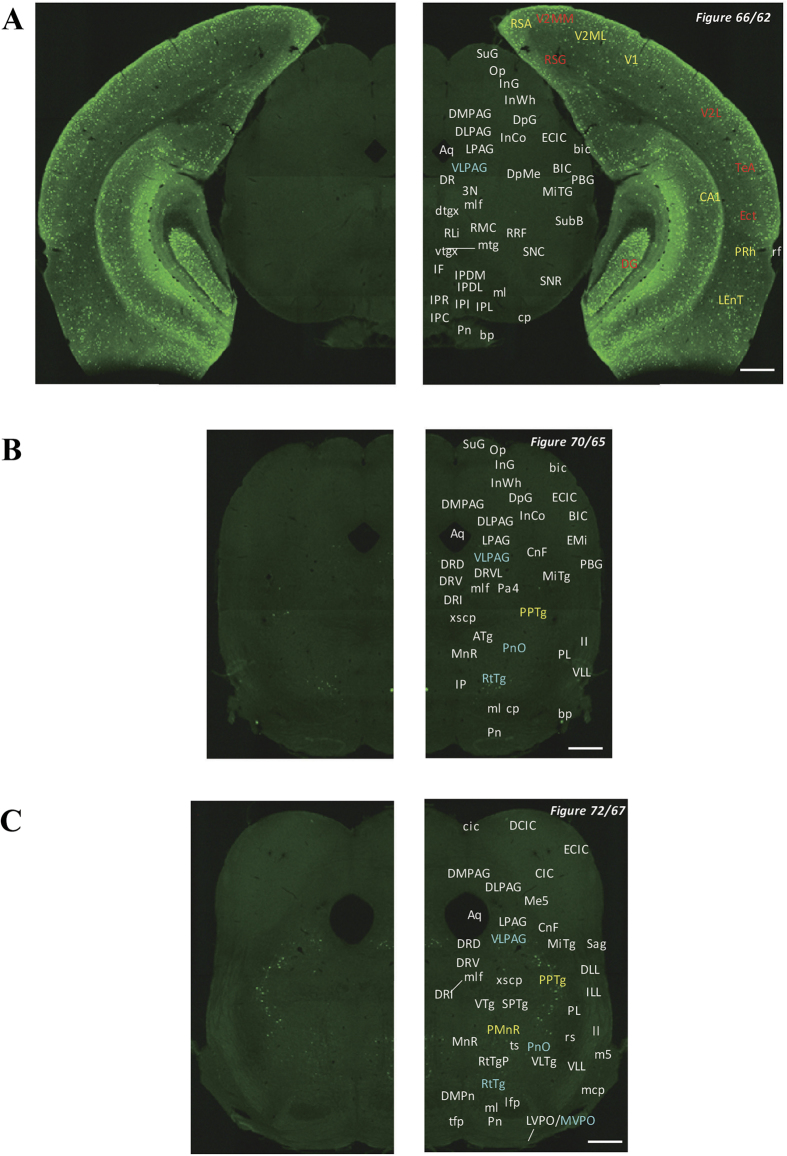
Detailed distribution of 5-HT3AR-expressing cells in the middle midbrain. The series of GFP immunofluorescent staining images of coronal sections of 5-HT3AR-TG mice from 62 to 72 (after-mentioned atras figure); Fluorescence images (left) and the abbreviation-tagged images (right); each abbreviation is linked to Table 1 and is coloured depending on the expression intensity: strong = red; moderately high = yellow; weak = blue; negative = white. The figure number in the upper right refers to two brain atlases (the Mouse Brain in Stereotaxic Coordinates 2nd edition and the Atlas of the Spinal Cord: Mouse, Rat, Rhesus, Marmoset, and Human). Scale bar: 500 µm.

**Figure 10:**
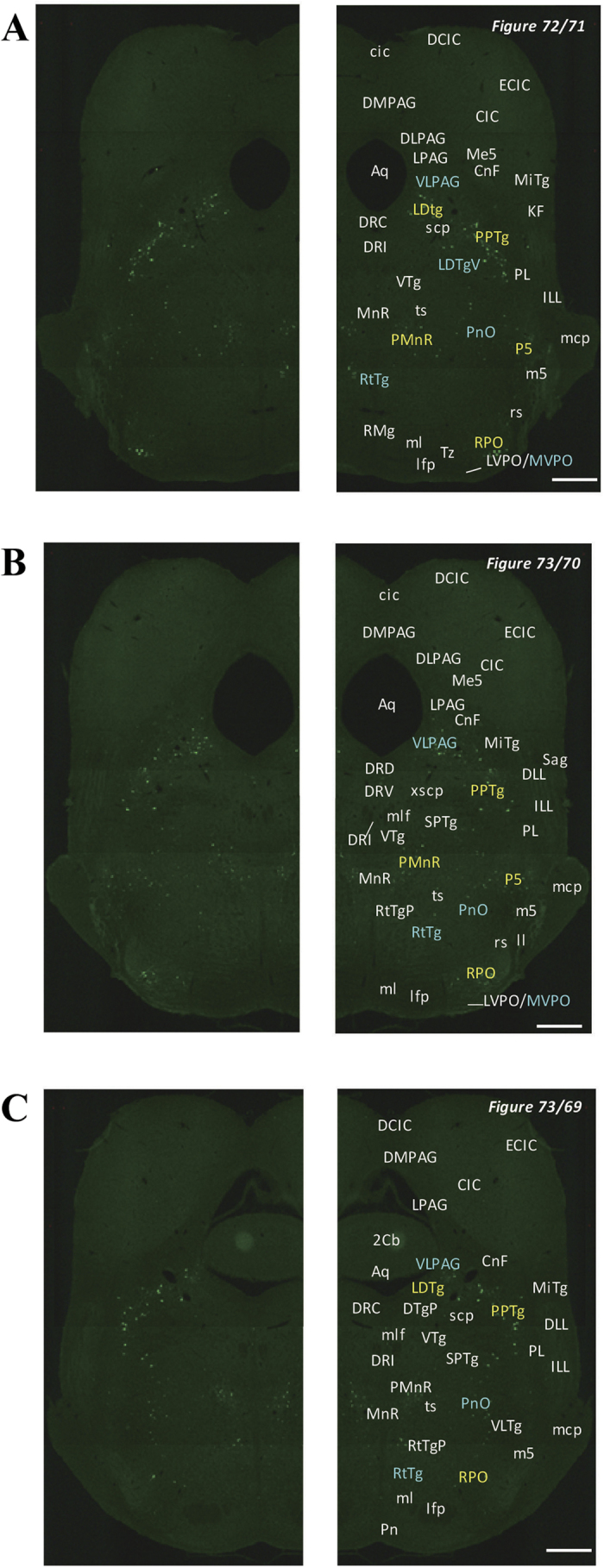
Detailed distribution of 5-HT3AR-expressing cells in the caudal midbrain and rostral pons. The series of GFP immunofluorescent staining images of coronal sections of 5-HT3AR-TG mice from 69 to 73 (after-mentioned atras figure); Fluorescence images (left) and the abbreviation-tagged images (right); each abbreviation is linked to Table 1 and is coloured depending on the expression intensity: strong = red; moderately high = yellow; weak = blue; negative = white. The figure number in the upper right refers to two brain atlases (the Mouse Brain in Stereotaxic Coordinates 2nd edition and the Atlas of the Spinal Cord: Mouse, Rat, Rhesus, Marmoset, and Human). Scale bar: 500 µm.

**Figure 11:**
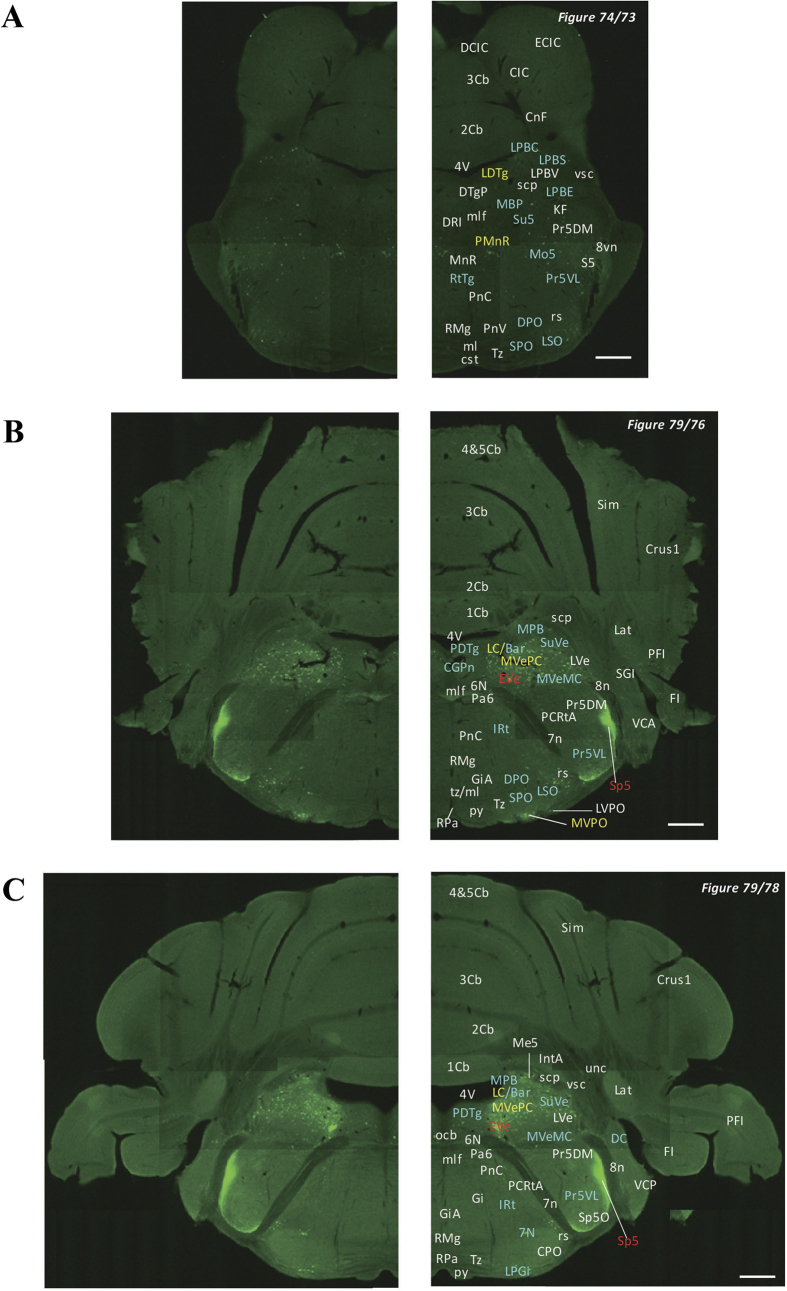
Detailed distribution of 5-HT3AR-expressing cells in the rostral pons and cerebellum. The series of GFP immunofluorescent staining images of coronal sections of 5-HT3AR-TG mice from 73 to 79 (after-mentioned atras figure); Fluorescence images (left) and the abbreviation-tagged images (right); each abbreviation is linked to Table 1 and is coloured depending on the expression intensity: strong = red; moderately high = yellow; weak = blue; negative = white. The figure number in the upper right refers to two brain atlases (the Mouse Brain in Stereotaxic Coordinates 2nd edition and the Atlas of the Spinal Cord: Mouse, Rat, Rhesus, Marmoset, and Human). Scale bar: 500 µm.

**Figure 12:**
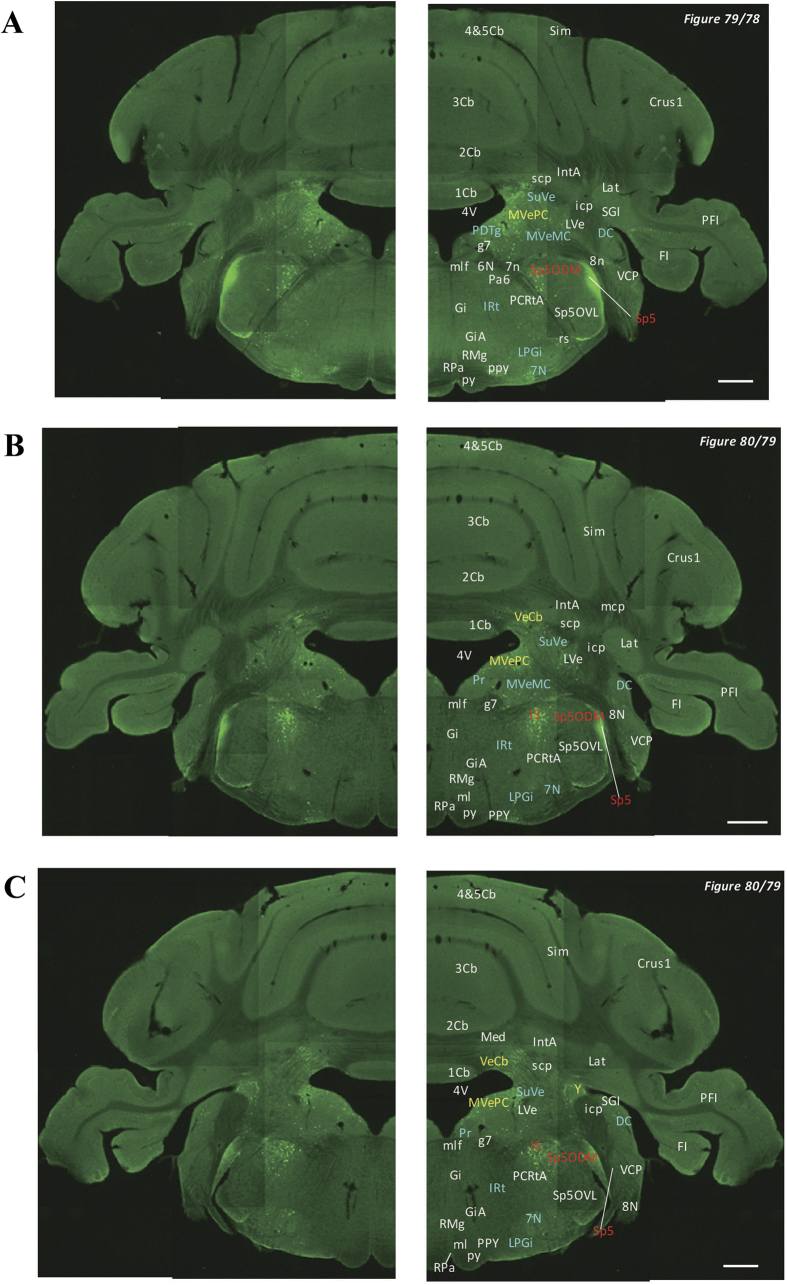
Detailed distribution of 5-HT3AR-expressing cells in the middle pons and cerebellum. The series of GFP immunofluorescent staining images of coronal sections of 5-HT3AR-TG mice from 78 to 79 (after-mentioned atras figure); Fluorescence images (left) and the abbreviation-tagged images (right); each abbreviation is linked to Table 1 and is coloured depending on the expression intensity: strong = red; moderately high = yellow; weak = blue; negative = white. The figure number in the upper right refers to two brain atlases (the Mouse Brain in Stereotaxic Coordinates 2nd edition and the Atlas of the Spinal Cord: Mouse, Rat, Rhesus, Marmoset, and Human). Scale bar: 500 µm.

**Figure 13:**
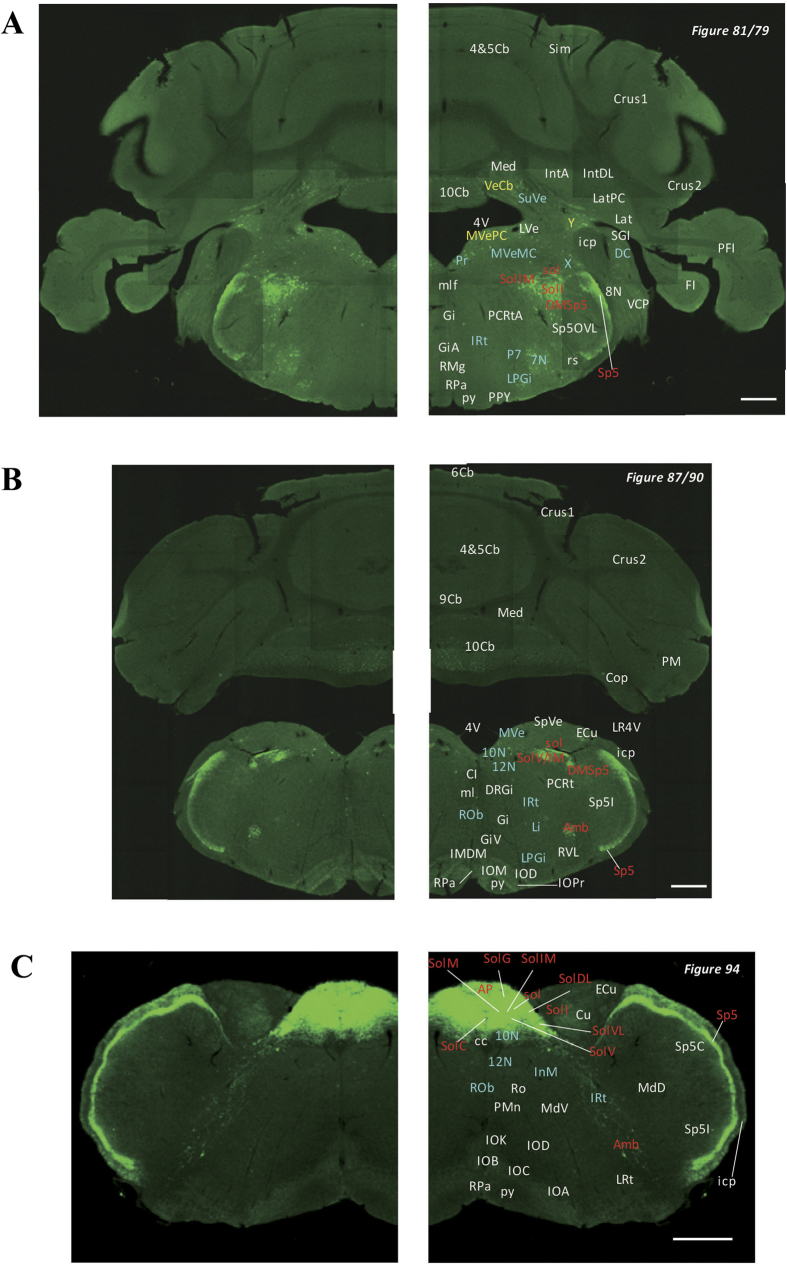
Detailed distribution of 5-HT3AR-expressing cells in the caudal pons, medulla oblongata and cerebellum. The series of GFP immunofluorescent staining images of coronal sections of 5-HT3AR-TG mice from 79 to 94 (after-mentioned atras figure); Fluorescence images (left) and the abbreviation-tagged images (right); each abbreviation is linked to Table 1 and is coloured depending on the expression intensity: strong = red; moderately high = yellow; weak = blue; negative = white. The figure number in the upper right refers to two brain atlases (the Mouse Brain in Stereotaxic Coordinates 2nd edition and the Atlas of the Spinal Cord: Mouse, Rat, Rhesus, Marmoset, and Human). Scale bar: 500 µm.

**Figure 14:**
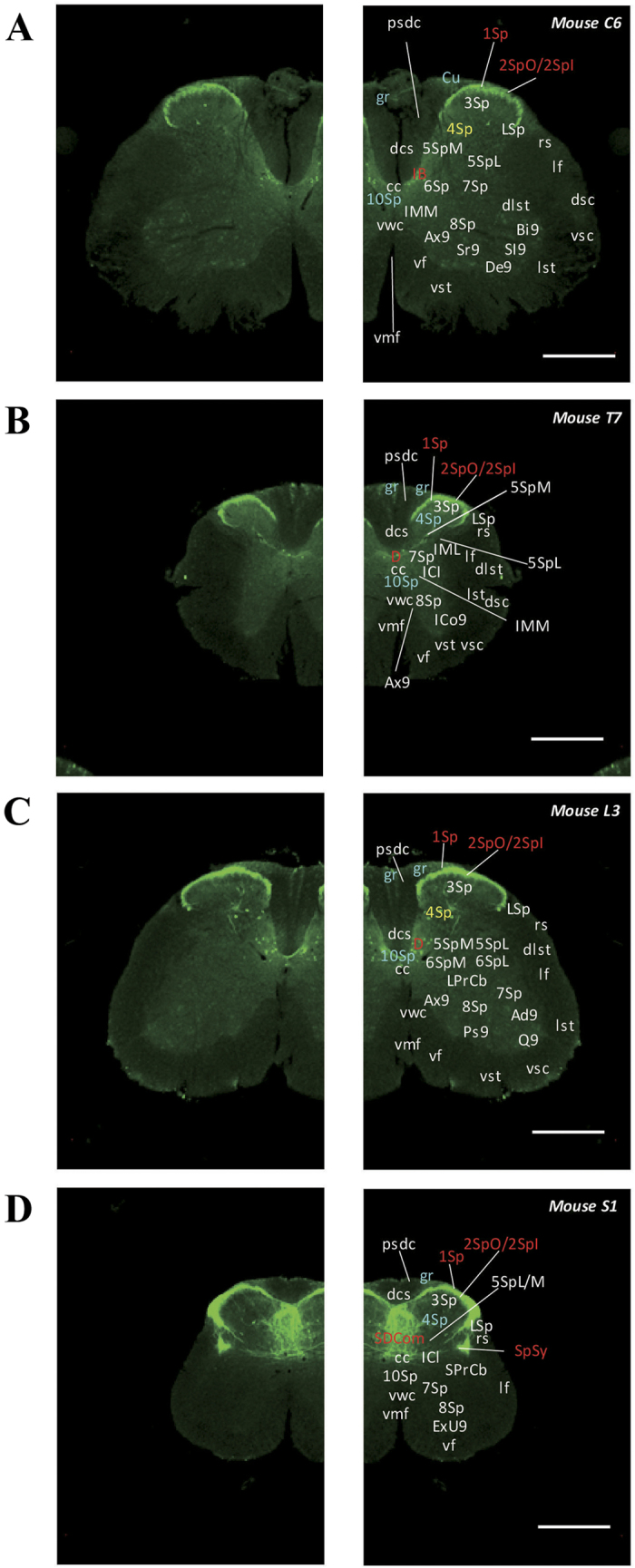
Detailed distribution of 5-HT3AR-expressing cells in the spinal cord. The series of GFP immunofluorescent staining images of coronal sections of 5-HT3AR-TG mice from Mouse C6 to S1 (after-mentioned atras figure); Fluorescence images (left) and the abbreviation-tagged images (right); each abbreviation is linked to Table 1 and is coloured depending on the expression intensity: strong = red; moderately high = yellow; weak = blue; negative = white. The figure number in the upper right refers to two brain atlases (the Mouse Brain in Stereotaxic Coordinates 2nd edition and the Atlas of the Spinal Cord: Mouse, Rat, Rhesus, Marmoset, and Human). Scale bar: 500 µm.

In the 5-HT3AR-GFP TG mice, we investigated the GFP-expressing cells throughout the CNS from the olfactory bulb to the sacral cord. They were distributed unevenly and showed various expression-based signal intensities depending on the region of the brain or spinal cord. The results of the distribution of 5-HT3AR-GFP-expressing cells are presented in Fig. 2 (overview) and Figs 3, 4, 5, 6, 7, 8, 9, 10, 11, 12, 13, 14 (individual images). In addition, the relative intensities of the GFP signals in each region of the brain and spinal cord are summarized in Supplementary Table 1. We categorized CNS as follow based on anatomical classification: Olfactory bulb and the forebrain olfactory system, Cerebrum (Cerebral cortex, Cerebral limbic system, Basal ganglia and Other regions of the forebrain), Diencephalon (Epithalamus, Thalamus and Hypothalamus), Brainstem (Midbrain, Pons, Medulla, Trigeminal nucleus and Raphe nucleus), Cerebellum, Spinal cord and Others.

### Olfactory bulb and the forebrain olfactory system

**Figure 15:**
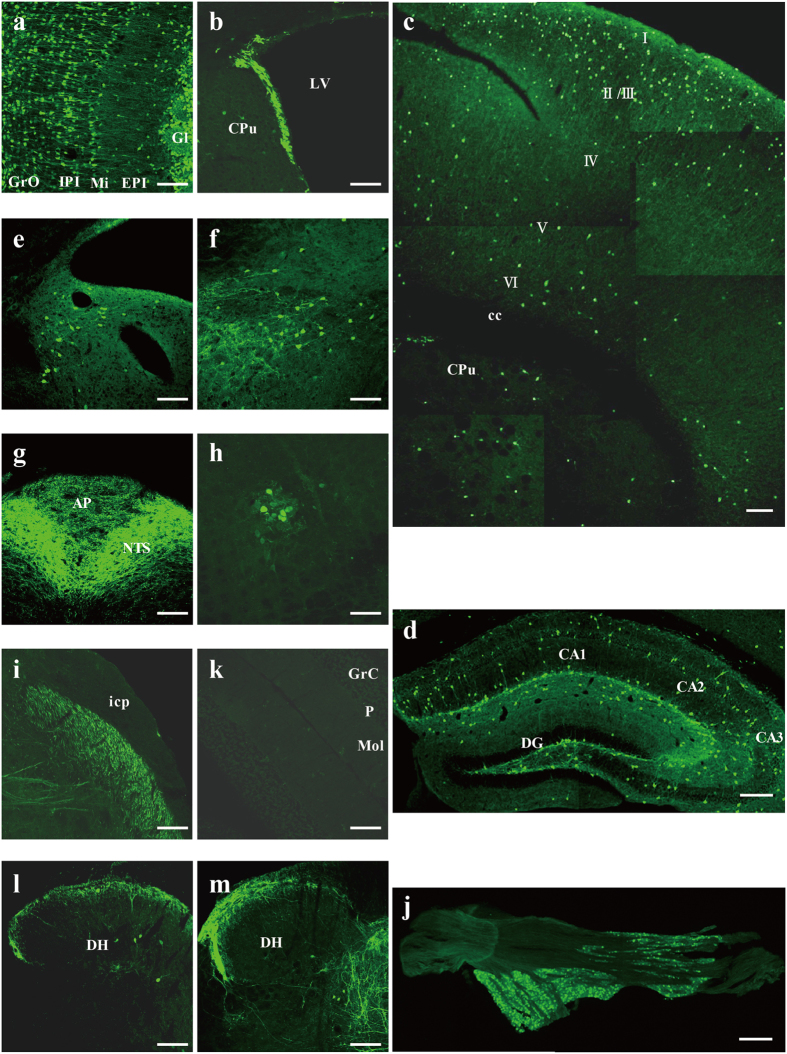
Microscopy under high power using 5-HT3AR-GFP TG mice. High-powered micrographs for GFP immunofluorescent staining of the olfactory bulb (**a**), lateral ventricle (**b**), somatosensory cortex (**c**), hippocampal formation (**d**), parvocellular medial vestibular nucleus (**e**), inferior salivatory nucleus (**f**), dorsal medulla containing the nucleus of the solitary tract, solitary tract, and area postrema (**g**), nucleus ambiguus (**h**), spinal trigeminal tract (**i**), trigeminal ganglion (**j**), cerebellum (**k**), the dorsal horn of the lumbar spinal cord, and (**l**) the dorsal horn of the sacral spinal cord (**m**). Each abbreviation is linked to Supplementary Table 1. (**c**) I, II/III, IV, V, VI: cell layer, (**k**) GrC: granular layer of cerebellum; P: Purkinje cell layer; Mol: molecular layer and (**l,m**) DH: spinal dorsal horn. Scale bar: 100 μm.

Many cells and nerve fibres in the olfactory bulb were labelled very strongly (Fig. 3a,b). Signals in the olfactory bulb were strong compared to those in other brain areas (Fig. 3c). Strongly labelled cells were observed in different layers of the olfactory bulb (i.e., mitral layer, granular layer, and periglomerular layer), but not in the accessory olfactory bulb (weak) or plexiform layer (moderately high) (Fig. 15a). Moreover, strong signals were observed in the subventricular zone from the lateral ventricle to the olfactory ventricle (Figs 3e, 4, 5, 6c and 15b). In the anterior olfactory nucleus, moderate signals were detected in most areas, while weak signals were seen in the ventral part (Fig. 3c,d). Signals were also detected in the olfactory tubercle, and much labelling was observed especially in the islands of Calleja (Fig. 3e). The nucleus of the lateral olfactory tract was moderately labelled (Fig. 6c).

### Cerebrum

Motor cortex/Cerebral cortex

The motor cortex was as strongly labelled as the somatosensory cortex (Figs 3e, 4, 5, 6c). The signals in the primary motor cortex were stronger than those in the secondary motor cortex.

**Somatosensory cortex/Sensory cortex/Cerebral cortex**

Somatosensory cortex: Strong or moderately high signals were detected in all regions of the somatosensory cortex (Figs 3e, 4, 5, 6, 7b). In particular, the barrel fields, forelimb, hindlimb, and dysgranular regions of the primary somatosensory cortex were strongly labelled. GFP-positive cells in all layers were scattered, and layer 2/3 was labelled more than the other layers (Fig. 15c).

**Auditory cortex and visual cortex/Sensory cortex/Cerebral cortex**

Many signals were detected in the auditory cortex (Figs 7c and 8c). In particular, the ventral area of the secondary auditory cortex was labelled strongly. On the other hand, there was a significant difference among the areas of visual cortex (Figs 7c, 8, 9a). The mediomedial and lateral areas of the secondary visual cortex were labelled strongly, while the mediolateral area of the secondary visual cortex and the primary visual cortex were labelled weakly.

**Insular cortex/Sensory cortex/Cerebral cortex**

Both the agranular and dysgranular insular cortices were strongly labelled, while the granular insular cortex was moderately labelled (Figs 3d, 4, 5, 6, 7b).

**Association cortex/Association cortex/Cerebral cortex**

Association cortex: There were many positive cells in the association cortex (Figs 7a, 8, 9a). Posterior areas (i.e., parietal association cortex and temporal association cortex) were more strongly labelled than the anteriorly located frontal association cortex.

**Prefrontal cortex/Association cortex/Cerebral cortex**

GFP signals were detected in all parts of the orbital cortex (Fig. 3c,d). While most signals detected were moderately high, the lateral orbital cortex was weakly labelled. Strong signals from both somata and neural fibres were observed in the following regions along the midline: prelimbic cortex, infralimbic cortex, and dorsal peduncular cortex (Figs 3d and 4b).

**Piriform cortex/Paleocortex/Cerebral limbic system**

Piriform cortex: Only weak signals were consistently detected in the piriform cortex and endopiriform nucleus (Figs 3d, 4, 5, 6, 7, 8a).

**Olfactory lobe/Paleocortex/Cerebral limbic system**

While signals were observed in many cells of the lateral entorhinal and perirhinal cortices, there was strong labelling in the ectorhinal cortex (Figs 7c and 8a).

**Hippocampus and dentate gyrus/Archicortex/Cerebral limbic system**

Although all areas of the hippocampus were labelled, signals were hardly detectable in the pyramidal and granular layers (Figs 6d, 7, 8, 9a). Many positive cells were observed in the molecular layers of the CA1, CA2, and CA3 regions. The nerve fibres in the radiate and oriens layers of the CA3 region were stained more than other areas (Fig. 15d). The somata and neural fibres in the polymorphic layer of the dentate gyrus were strongly labelled, whereas no signals were detected in the molecular layer (Fig. 15d).

**Cingulate cortex/Mesocortex/Cerebral limbic system**

Many positive cells were consistently observed in the cingulate cortex from the rostral (cingulate cortex) to the caudal region (retrosplenial cortex) (Figs 3d, 4, 5, 6, 7, 8, 9a). Interestingly, the ventral parts (i.e., cingulate cortex area 2 and retrosplenial granular cortex) exhibited strong signals in neural fibres and somata.

**Amygdala/Subcortical nuclei/Cerebral limbic system**

All the amygdaloid subnuclei and regions were labelled, and strong and moderately high signals were observed everywhere except in the ventral part of the anterior amygdaloid area and the intercalated cells (Figs 6b, 7, 8c). In particular, the basolateral amygdaloid nucleus (BLA), posterior cortical amygdaloid nucleus (PlCo), posterior part of the basomedial amygdaloid nucleus, and amygdalohippocampal area were strongly labelled.

**Bed nucleus of the stria terminalis/Subcortical nuclei/Cerebral limbic system**

Signals were detected in all areas of the bed nucleus of the stria terminalis (Figs 5c, 6, 7, 8, 9a). Interestingly, the upper and lower sides of the anterior commissure differed in their signal intensities. The upper regions were labelled more strongly than the lower regions; this difference was larger between the posterior and anterior parts. In particular, the supracapsular part and the medial division (i.e., posteromedial and posterointermediate parts) of the bed nucleus of the stria terminalis were labelled quite strongly (Figs 6b and 7a).

**Septum/Subcortical nuclei/Cerebral limbic system**

Signals were hardly detectable in the septum (Figs 4c and 5c). GFP signals were barely observed in the lambdoid septal zone or the septohippocampal and septofimbrial nuclei.

**Mammillary bodies/Subcortical nuclei/Cerebral limbic system**

GFP signals were hardly observed in the mammillary bodies (Fig. 8a–b). Weak signals were detected in the medial part of the supramammillary nucleus as well as the medial and median parts of the medial mammillary nucleus.

### Basal ganglia

In the basal ganglia, there was strong labelling in the claustrum, moderately high labelling in the caudate and putamen (CPu) and the shell of the nucleus accumbens, and weak labelling in the core of the nucleus accumbens (Figs 3e, 4, 5a). No signals were detected in the nucleus basalis of Meynert (Fig. 6c), the globus pallidus (Figs 6a and 7b), or the substantia nigra (Figs 8a and 9a).

### Other regions of the forebrain

In the diagonal band of Broca, both the nucleus of the vertical limb (Figs 4b and 5a) and the nucleus of the horizontal limb (Figs 5c and 6b) were weakly labelled. The semilunar nucleus of Flechsig was also weakly labelled (Fig. 3e). Signals were also detected in the tenia tecta, where the dorsal parts were labelled more strongly than the ventral parts.

## Diencephalon

**Epithalamus**

No signal was detected in all the areas and nuclei of the epithalamus, including the habenular nuclei (Figs 6b and 7b).

Thalamus

Anterior, lateral, and ventrolateral nucleus group.

No signal was detected in all anterior, lateral, and ventrolateral nuclei of the thalamus, including the various specific nuclei (Figs 6a and 7c).

**Anterior, lateral, and ventrolateral nucleus group/Thalamus**

No signals were detected in any specific medial nuclei of the thalamus, except for one non-specific nucleus, the posterior intralaminar thalamic nucleus, where the signals detected were weak (Figs 7a and 8c).

**Medial nucleus group/Thalamus**

No signals were detected in the posterior nucleus group of the thalamus, including the lateral and medial geniculate nuclei, except for in the suprageniculate thalamic nucleus, where the detected signals were very weak (Figs 7a and 8c).

**Preoptic area/Hypothalamus**

Weak signals were observed in the lateral preoptic nucleus (Fig. 5b), magnocellular preoptic nucleus (Figs 5c and 6c), and medial preoptic nucleus (Figs 6b and 7a).

**Medial zone/Hypothalamus**

In the supraoptic area, signals were observed in the paraventricular hypothalamic nucleus and the ventrolateral part of the suprachiasmatic nucleus. In particular, the posterior part of the paraventricular hypothalamic nucleus was moderately labelled (Fig. 7a,b). In the pars tuberalis, only the dorsomedial hypothalamic nucleus was labelled, and the signals in the compact part were stronger than those in other parts (Fig. 7c).

**Lateral zone/Hypothalamus**

No signal was detected in all the areas and nuclei of the lateral zone of the hypothalamus (Fig. 7b,c).

**Periventricular nucleus/Hypothalamus**

The periventricular nucleus was not labelled at all (Fig. 7b,c).

## Brainstem

**Midbrain**

No signals were detected in the midbrain except in the periaqueductal gray (PAG; rostral and ventrolateral parts; Figs 8a, 9, 10c), nucleus of Darkschewitsch (Figs 8a, 9, 10c), peripeduncular nucleus (PP; Fig. 8b,c), and the dorsal terminal nucleus of the accessory optic tract (Fig. 8b). Most detectable signals were very weak and only the rostral PAG was moderately labelled.

**Pons**

Signals in the pons were detected over a wide range, and various expression levels were observed.

**Ventral pons (basilar pons)/Pons**

Neither the pontine nucleus nor the trapezoid body was labelled (Figs 9a, 10a, 11a). Meanwhile, in the nucleus associated with superior olive, lateral superior olive (Fig. 11a,b), superior periolivary nucleus (Fig. 11a,b), and dorsal periolivary region (Fig. 11a,b) were labelled weakly, while the medioventral periolivary nucleus (Figs 9c and 10b) and rostral periolivary region (Fig. 10a–c) were moderately labelled.

**Vestibular nuclei, cochlear nuclei, and associated nuclei/Dorsal pons (pontine tegmentum)/Pons**

Signals were detected in the vestibular nuclei except for the spinal and lateral vestibular nuclei (Figs 11b, 12b, 13b). The efferent vestibular nucleus was labelled strongly (Fig. 11c). The parvocellular part of the medial vestibular nucleus (Figs 11b, 12, 13a and 15e) and the vestibulocerebellar nucleus (Figs 12b and 13a) were labelled moderately. The magnocellular part of the medial vestibular nucleus (Figs 11b and 12b), the superior vestibular nucleus (Figs 11b and 12c), and the medial vestibular nucleus were all weakly labelled (Fig. 3hh). The dorsal cochlear nucleus was weakly labelled (Figs 11c, 12, 13a). Nuclei X and Y were labelled at low and moderate levels, respectively (Figs 12c and 13a).

**Tegmental nucleus/Dorsal pons (pontine tegmentum)/Pons**

The pedunculopontine tegmental nucleus (Figs 9b and 10c) and laterodorsal tegmental nucleus (Figs 10c and 11a) were moderately labelled. The posterodorsal tegmental nucleus (Figs 11b and 12a), reticulotegmental nucleus of the pons (Figs 9b, 10, 11a), and the ventral laterodorsal tegmental nucleus were weakly labelled (Fig. 10a).

**Reticular, facial, abducens, and parabrachial nuclei/Dorsal pons (pontine tegmentum)/Pons**

Signals were observed in the reticular, facial, and parabrachial nuclei but not in the abducens nucleus. In particular, the parabrachial nuclei were weakly labelled aside from the ventral part of the lateral parabrachial nucleus (Fig. 11a,b). The facial nucleus (Figs 11c, 12, 13a), accessory facial nucleus (Fig. 13a), intermediate reticular nucleus (Figs 11b, 12, 13c), and oral part of the pontine reticular nucleus (Figs 9b and 10c) were also labelled.

**Others/Dorsal pons (pontine tegmentum) /Pons**

The inferior salivatory nucleus (IS) was strongly labelled (Figs 12b,c and 15f), and the locus coeruleus was moderately labelled (Fig. 11b,c). The Barrington’s nucleus (Fig. 11b,c), central gray of the pons (Fig. 11b), lateral paragigantocellular nucleus (Figs 11c, 12, 13b), and nucleus prepositus (Figs 12b and 13a) were weakly labelled.

## Medulla

The nucleus tractus solitarius (NTS), solitary tract (Fig. 15g), and nucleus ambiguus (Fig. 15h) were strongly labelled. Very strong signals were observed in all parts of the NTS (Fig. 13a–c). Meanwhile, weak signals were detected in the linear nucleus of the medulla (Fig. 13a,b), DMV (Fig. 13b,c), the hypoglossal nucleus (Fig. 13b,c), and the intermedius nucleus of the medulla (Fig. 13c).

## Trigeminal nucleus

Signals were observed throughout the trigeminal nucleus except in the mesencephalic trigeminal nucleus (Figs 9c and 10b). In particular, the oral part of the spinal trigeminal nucleus (dorsomedial division; Fig. 12a–c), the dorsomedial spinal trigeminal nucleus (Fig. 12a–c), and Sp5 (Figs 11b, 12, 13c and 15j) were strongly labelled. The peritrigeminal zone (Fig. 10a,b) was moderately labelled, and the motor trigeminal nucleus (Fig. 11a), the ventrolateral part of the principal sensory trigeminal nucleus (Fig. 10a,b), and the supratrigeminal nucleus (Fig. 11a) were weakly labelled. Furthermore, strong signals were detected in many cell bodies in the trigeminal ganglion (Fig. 15j).

## Raphe nucleus

There were no signals in the raphe nuclei except for in the paramedian raphe nucleus (Fig. 10a,b) and raphe obscurus nucleus (Fig. 13b,c). The signals in the paramedian raphe nucleus were clear and moderate, while the signals in the raphe obscurus nucleus were weak.

## Cerebellum

No signals were observed in main cerebellar cortex neurons such as Purkinje cells, granular cells, satellite cells, basket cells, and Golgi cells (Figs 11b, 12, 13b and 15k). In addition, GFP signals were not observed in any cerebellar nuclei (i.e., interposed cerebellar nucleus, dentate cerebellar nucleus, and fastigial cerebellar nucleus).

## Spinal cord

Strong signals were detected in lamina 1 and 2 of the spinal cord (Figs 14a–d and 15l,m). Moreover, signals were also observed in lamina 4 and their intensity varied in different areas. Signals were moderately high in cervical and lumbar areas and weak in the thoracic and sacral areas. In area 10 of the spinal gray matter, weak signals were observed along the length of the spinal cord except for in thoracic areas. Strong signals were also observed in the dorsal nucleus (Clarke’s nucleus; Fig. 14b,c) in the thoracic and lumbar areas. In the white matter, signals were observed in the gracile fasciculus (Fig. 14a–d) and cuneate fasciculus (Fig. 14a). In particular, patterns of signals in the sacral spinal cord were interesting (Figs 14d and 15n). The sacral parasympathetic nucleus and the sacral dorsal commissural nucleus were strongly labelled, and many labelled nerve fibres were observed along the intercalated nucleus areas.

## Others

### Circumventricular organs

In the sensory circumventricular organ, only the AP (Figs 13c and 15g) was labelled robustly. Neither the vascular organ of the lamina terminalis (Fig. 5b,c) nor the subfornical organ (Fig. 6a) were labelled. Meanwhile, signals in the median eminence (secretory circumventricular organ) were not detected at all.

### Fascicles of nerve fibres and associated nuclei

Some signals were detected in the commissural fibre (Figs 3e, 4, 5, 6b), whereas only weak signals were observed in the rubrospinal tract (Figs 11a and 12a). Signals were also observed in the fascicle of the anterior commissure and the corpus callosum (Figs 3e and 6b). We confirmed that these cells were neurons in the fascicle, as described in the results of our double immunofluorescence staining (Supplemental Figure 3). The anterior commissural nuclei (Fig. 6c) were weakly labelled, and the interstitial nuclei of the posterior limb of the anterior commissure (Figs 5c and 6a) were strongly labelled.

### Identification of cell types of 5-HT3AR-expressing cells

**Figure 16:**
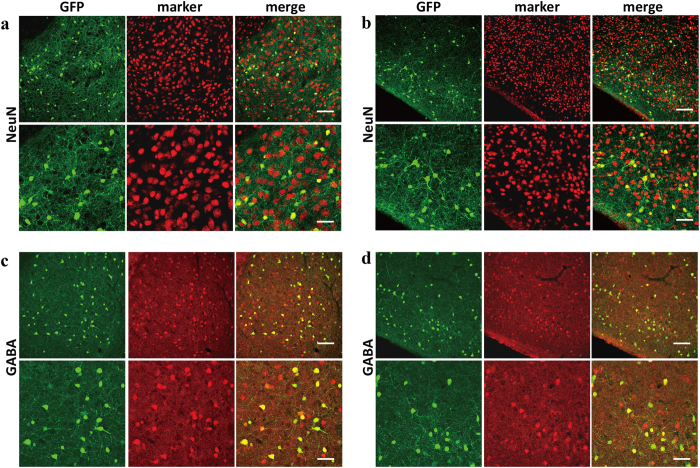
Identification of the 5-HT3AR-expressing cell types. The micrographs of double staining for the basolateral amygdaloid nucleus (**a,c**), posterior cortical amygdaloid nucleus (**b,d**) of 5-HT3AR-GFP TG mice using anti-NeuN antibody (**a,b**) and anti-GABA antibody (**c,d**). Magnification: low (upper) and high (lower). Fluorescent signals: left (GFP), middle (each marker), and right (merge). Scale bar: 100 ì m (upper) and 200 ì m (bottom).

Finally, to investigate the cell types of 5-HT3AR-expressing cells, we performed immunofluorescent staining with various cell markers as follows: NeuN for neuronal cells, GFAP for astroglia, APC for oligodendroglia, and CD11b (alias Integrin αM chain) for microglia. Many GFP signals were detected in the amygdala; the BLA and PlCo showed particularly strong signals (Fig. 7c). GFP expression was observed from the soma to the end of the neurites, while NeuN was detected in the nucleus. In both the BLA and PlCo, all the GFP-positive cells also expressed NeuN (Fig. 16a,b). Since DAPI is a nuclear marker, the GFP was colocalized with DAPI in cells with small nuclei (Supplemental Fig. 3C). On the other hand, the subcellular localizations of GFAP, APC, and CD11b were observed in the cytoskeleton, cytoplasm, and cell membrane, respectively, and were detected clearly in both the amygdaloid areas. However, GFP signals were not colocalized with the signals representing the glial markers (Supplemental Fig. 2A,B). We also confirmed that GFP was not expressed in both astrocytes and oligodendrocytes, as we did not find overlapping images in the corpus callosum and hippocampal CA3 region (Supplemental Fig. 3A,B). These results suggest that the GFP-expressing cells were neurons with small nuclei.

**Figure 17:**
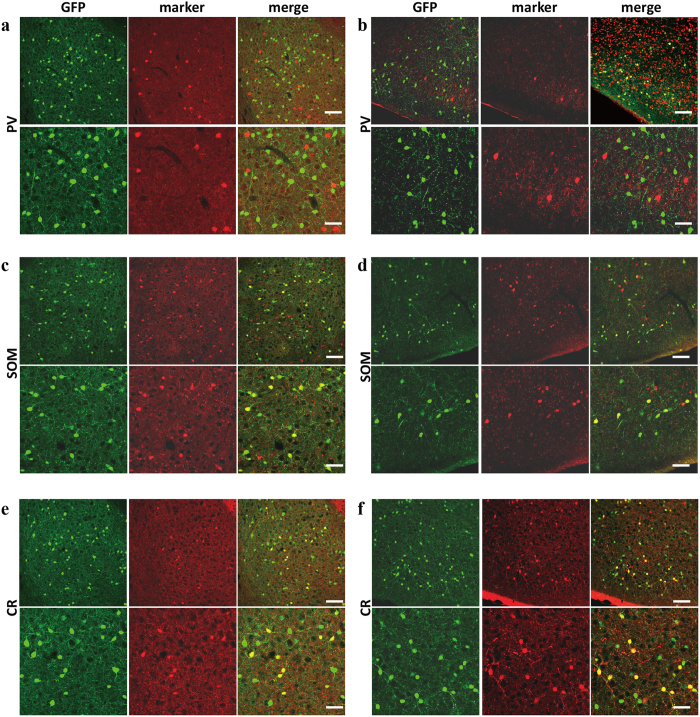
Identification of the 5-HT3AR-expressing GABAergic interneuron subtypes. The micrographs of double staining for the basolateral amygdaloid nucleus (**a,c,e**), posterior cortical amygdaloid nucleus (**b,d,f**) of 5-HT3AR-GFP TG mice using anti-parvalbumin antibody (**a,b**), anti-somatostatin antibody (**c,d**), anti-calretinin antibody (**e,f**). Magnification: low (upper) and high (lower). Fluorescent signals: left (GFP), middle (each marker), and right (merge). Scale bar: 100 μm (upper) and 200 μm (bottom).

Subsequently, the 5-HT3AR expression in the interneuron was examined using immunofluorescent staining for gamma-aminobutyric acid (GABA), a marker for GABAergic interneuron. As the result, almost all the 5-HT3AR-expressing neurons were identified as GABAergic interneurons (Fig. 16). Further, we also examined the kind of GABAergic interneurons expressed by the 5-HT3AR. On the basis of the neurochemical marker, GABAergic interneurons were divided into three main categories: parvalbumin (PV), somatostatin (SOM), and calretinin (CR)^42^. Immunofluorescence staining using the three antibodies listed above demonstrated that 5-HT3AR-expressing interneurons overlapped with SOM-positive and CR-positive interneurons, but not with PV-positive interneurons (Fig. 17). As for the SOM and CR, the rates of co-positive staining based on GFP were 44.58 ± 0.04% (SOM, BLA), 45.56 ± 0.04% (SOM, PlCo), 21.78 ± 0.02% (CR, BLA), and 20.62 ± 0.02% (CR, PlCo). The 5-HT3AR expressed in GABAergic interneurons containing SOM or CR in the BLA and PlCo.

## Discussion

In the present study, we developed a complete and detailed distribution map of 5-HT3AR in the CNS of mice. 5-HT3AR was expressed in GABAergic interneurons containing somatostatin or calretinin, and most strongly in the olfactory bulb, cerebral cortex, hippocampus, and amygdala. The pons, medulla, and spinal cord showed partial 5-HT3AR expression. Meanwhile, slight expression of 5-HT3AR was observed in the thalamus, hypothalamus, and midbrain; no expression was seen in the cerebellum. Thus, the expression pattern of 5-HT3AR is unique and widespread throughout the CNS.

Receptor autoradiography and immunohistochemistry have been successfully used in studies investigating the distribution of 5-HT3AR. Radioligand binding studies with various highly-specific drugs have disclosed the localization of 5-HT3R in the brains of many species ranging from mice to humans. However, there has been somewhat of a controversy because the binding sensitivity varied among the selected ligands. Although dense binding was observed in the hindbrain, especially in the NTS, regardless of the species, the results in other regions such as the DMV and Sp5 were different depending on the drugs used. For example, the radioligand binding in the Sp5 was low or nonexistent when either [3H]-GR65630 or [3H]-granisetron was used, while binding using [3H]-zacopride or [3H]-ICS205930 was strong in the same area^43^. In addition, there were major differences in the results in the AP among the selected drugs (strong signals using [3H]-tropisetron and [3H]-LY278584^9^,^19^; low signals using [3H]-granisetron^17^,^44^,^45^). Moreover, findings obtained from radiographic analyses were inadequate for understanding detailed localization in the subnuclei. As distributional studies with autoradiography have significant limitations, they cannot provide consistent and detailed information. On the other hand, immunohistochemical analysis enables us to acquire much more detailed information, such as subcellular localization and subnuclear localization. Furthermore, unlike selective drugs, antibodies for 5-HT3R were able to specifically bind to the 5-HT3AR subunit. Starting with those used in the study by Turton *et al*., many antibodies to 5-HT3R have been produced^29^,^38^,^46^,^47^,^48^ and have uncovered the localization of 5-HT3R in the CNS^28^,^29^,^31^,^32^,^33^,^39^,^49^. Three kinds of antibodies specific for 5-HT3R have been employed: antibodies against (1) 444–457 amino acids of the second cytoplasmic loop regions^38^; (2) 375–395 amino acids of the second cytoplasmic loop regions^48^; (3) 23–36 amino acids of the exodomain^29^. Unfortunately, the patterns of subcellular distribution were dependent on the respective epitopes of the 5-HT3R antibodies. The antibodies recognized (1) the perikaryon and dendrites, (2) the axon and nerve terminals, (3) the soma, dendrites, and axons. As these differences had the potential to affect the interpretations of the results, it had been difficult to obtain a more specific expression map of 5-HT3R. Considering the cerebellum, some studies have reported expression of 5-HT3R^30^,^50^, while others have shown no expression^28^,^31^,^48^. In order to investigate the distribution of 5-HT3R in the CNS more precisely and extensively, we used 5-HT3AR-GFP TG mice purchased from the Mutant Mice Regional Resource Center^40^,^41^. Since GFP staining was observed in whole neurons, including the axons and dendrites, 5-HT3AR-GFP TG mice were not suitable for the study of its intracellular localization. However, it is good to examine the relationship between 5-HT3AR and CNS neural networks. Nevertheless, distributional studies using 5-HT3AR-GFP TG mice were restricted to certain regions, such as the cerebral cortex^40^, hippocampal formation^40^, subventricular zone^51^,^52^, and olfactory bulbs^53^. Therefore, in this study, we went into the fine details of the distribution of 5-HT3AR throughout the CNS from the olfactory bulb to the sacral spinal cord and obtained a detailed expression map for 5-HT3AR (Supplementary Table 1).

We investigated whether 5-HT3AR actually expressed in the GFP-positive cells by performing 5-HT3AR expression analysis using 5-HT3AR-GFP TG mice. *In situ* hybridization using mirror image sections (mirror-ISH) were well suited to examine the expression of two factors in the same cell because the staining results were unaffected by the cross-hybridization between the two probes and the overlap between their wavelengths. Mirror-ISH were highly specific methods to examine expression levels in an independent reaction system. However, this method has one weakness, in that the co-positive rate of perfect matching for mirror-ISH cannot be 100% because of the loss of cross-section surface and the imbalanced volumes of divided cells contained in both sections (left and right). In fact, when mirror-ISH with the exact same probe (5-HT3AR) were performed, the co-positive rate of using the exact same probe was 57.2 ± 4.7% and did not reach 100% (Supplemental Fig. 1A–D). Therefore, approximately 60% was used as the co-positive rate of perfect matching for mirror-ISH. On the other hand, the co-positive rates of mirror-ISH using the GFP and 5-HT3AR probes were 57.2 ± 2.6% (GFP) and 67.8 ± 3.2% (5-HT3AR) (Supplemental Fig. 1E). Because the co-positive rates of GFP and 5-HT3AR were nearly the same as that of the standard, we considered that GFP and 5-HT3AR were expressed in the same cell.

Given that 5-HT3R antagonists have been reported to show antiemetic effects^54^,^55^,^56^,^57^,^58^, 5-HT3R expression in brain areas responsible for vomiting including the NTS, DMV, and AP, has been well examined^8^,^9^,^11^,^13^,^24^,^43^,^59^,^60^. In accordance with previous reports, the present study also shows high expression of 5-HT3R in areas associated with vomiting. Moreover, as the activation of 5-HT3R is relevant for pain perception, the expression of 5-HT3R in the dorsal horn of the spinal cord has also been well investigated^61^,^62^,^63^,^64^,^65^,^66^,^67^,^68^,^69^,^70^,^71^,^72^. Our data showing strong expression of 5-HT3R in the superficial layers of the dorsal horn of the spinal cord are consistent with data from previous studies^13^,^26^,^28^,^31^,^48^,^60^,^73^.

In addition, our analysis also shows strong expression of 5-HT3AR in the amygdala, hippocampal formation, cerebral cortex, and olfactory system including the olfactory bulb. Our findings are consistent with several previous findings about the amygdala^9^,^11^,^18^,^20^,^22^,^23^,^26^,^28^,^32^,^33^,^60^,^74^, hippocampal formation^9^,^11^,^15^,^20^,^22^,^23^,^24^,^26^,^74^, cerebral cortex^8^,^9^,^11^,^13^,^20^,^22^,^24^,^26^,^28^,^32^,^33^,^39^,^59^,^60^, and the olfactory system (olfactory bulb^26^,^62^; accessory olfactory nucleus^28^,^60^; olfactory tubercle^33^; olfactory lobe^26^,^31^,^33^). Remarkably, the patterns of strong expression in the subareas of certain regions are also consistent with those observed in previous reports: the BLA and the cortical nucleus of the amygdala^32^, CA3 and DG of the hippocampal formation^15^,^24^,^33^, cortical layer 2/3^26^,^28^,^39^,^60^, and the glomerular layer of the olfactory bulb^26^,^60^. Notably, our analysis revealed that the amygdalohippocampal area is a novel amygdaloid subarea in which 5-HT3R is strongly expressed. Moreover, a closer examination also provided new findings of the distribution of 5-HT3R in subareas of the cortex. For instance, previous studies have reported high expression of 5-HT3R in the cingulate cortex^11^,^20^,^21^,^28^,^33^,^60^. In contrast, in the present study, we found that expression levels in the cingulate cortex differed between subareas; they were moderately high in area 1 and strong in area 2. Interestingly, the differences in the expression levels of 5-HT3R among the subareas were also observed within the same cortical regions including the motor cortex, insular cortex, somatosensory cortex, visual cortex, and auditory cortex. Moreover, we found strong expression of 5-HT3R in other regions such as the tenia tecta^28^,^60^, bed nucleus of the stria terminalis^33^, corpus callosum^52^, Sp5^15^,^26^,^27^,^28^,^31^,^48^.

Fortunately, the present study identified distinct regions and nuclei in which 5-HT3R was expressed intensely, such as the efferent vestibular nucleus, IS, and nucleus ambiguus. In addition, we have successfully identified many new areas in which moderately high expression of 5-HT3R was observed, such as the interstitial nuclei of the posterior limb of the anterior commissure, medioventral periolivary nucleus, vestibulocerebellar nucleus, and locus coeruleus. Notably, many regions showing high expression of 5-HT3R in the pons (e.g., IS, locus coeruleus, efferent vestibular nucleus, and medioventral periolivary nucleus) were identified. As there were few observations of 5-HT3R expression in the pons except for in a few areas (i.e., tegmental nucleus and facial nucleus)^26^,^28^, our findings are quite important. In addition, previous reports have made little mention of the expression of 5-HT3R in the raphe nuclei; only the raphe obscurus nucleus showed a weak expression level^27^, while the other raphe nuclei including the raphe magnus nucleus^27^ and the dorsal raphe nucleus^59^ showed no 5-HT3R expression. In addition to the low expression in the raphe obscurus nucleus, we found moderately high expression in the paramedian raphe nucleus. Furthermore, although 5-HT3R expression was hardly detectable in the thalamus and hypothalamus, consistent with previous studies^22^,^24^,^28^ except in hamster brains^33^, we found two subareas in which the 5-HT3R was expressed strongly in the hypothalamus, dorsomedial hypothalamic nucleus, and paraventricular hypothalamic nucleus.

On the other hand, the piriform cortex, CPu, nucleus accumbens, and cerebellum were controversial areas regarding the expression pattern of 5-HT3R. In particular, the literature is split on the expression levels of 5-HT3R in the piriform cortex and CPu: piriform cortex, strong^26^,^28^,^33^,^60^ or weak^31^,^48^; CPu, strong^15^,^28^ or weak^23^,^24^. Therefore, our results (piriform cortex, weak; CPu, moderate) provide further evidence of the possible roles of 5-HT3R. Moreover, the lack of expression of 5-HT3R in the cerebellum is consistent with previous reports^3^,^15^,^22^,^28^,^60^. Meanwhile, a few previous studies reported 5-HT3R expression at synapses within the cerebellum^30^,^50^. However, no signals were found in our thorough observation of the cerebellum. In the nucleus accumbens, our findings are in agreement with previous studies showing that 5-HT3R expression in the shell of the nucleus accumbens is much stronger than that in the core^15^,^33^, except in the study by Morales and colleagues^28^. Thus, the present study using 5-HT3AR-GFP TG mice revealed that high 5-HT3AR expression was observed in the CPu and the shell of the nucleus accumbens. In addition, strong expression of 5-HT3R was observed in the claustrum in conformity with previous studies^28^,^60^, and there was no expression in the substantia nigra, basal nucleus of Meynert, or globus pallidus. The variety of expression patterns raises the possibility of different, specific roles of 5-HT3R in the basal ganglia. In the midbrain, 5-HT3R expression was moderate in the PAG and weak in the nucleus of Darkschewitsch, the peripeduncular nucleus, and the dorsal terminal nucleus of the accessory optic tract. Expression sites in the midbrain remain a matter of debate in the literature as follows: little detection in the midbrain^60^, PAG^33^, trochlear nerve^26^,^28^, red nuclei^28^,^33^, oculomotor nuclei^28^, superior colliculus^28^,^33^, inferior colliculus^28^, and deep mesencephalic nuclei^33^. Our analysis shows that there was little detection in the midbrain except in the PAG, which is in agreement with Gehlert’s and Carrillo’s findings^33^,^60^. However, as the dorsal terminal nucleus of the accessory optic tract branches from the medial roots of the brachium of the superior colliculus, and the nucleus of Darkschewitsch projects to the oculomotor nuclei in part, the expression of 5-HT3R in the superior colliculus and oculomotor nuclei is also consistent with that observed in previous reports. No expression was observed in the red nuclei or the trochlear nerve at all in the present study.

Finally, we examined the 5-HT3AR-expressing cell types in the amygdala, where 5-HT3AR-mediated neurotransmission plays a significant role in emotional function^75^,^76^,^77^,^78^. Our results confirmed that 5-HT3AR was expressed in neurons but not in glia, which is in agreement with previous findings^28^,^29^,^30^,^31^,^32^,^33^,^40^,^51^,^52^,^53^. Many previous reports have shown that 5-HT3Rs are expressed in interneurons in the cortex^52^,^79^, amygdala^32^,^79^, olfactory bulb^51^,^53^, hippocampus^26^,^40^,^79^, and spinal cord^80^,^81^. On the other hand, Morales *et al*. reported that 5-HT3R was expressed in two kinds of neurons: interneurons and motor neurons^28^. They suggested that the 5-HT3R-expressing motor neurons were located in the motor trigeminal nucleus, facial nucleus, hypoglossal nucleus, and ventral horn of the spinal cord. Other studies have also reported 5-HT3R expression in these areas^26^,^27^. However, in the present study, the expression of 5-HT3AR in these regions was very weak. Moreover, our data revealed that 5-HT3AR was expressed by the GABAergic interneurons. Therefore, we suggest that most 5-HT3R-expressing neurons were GABAergic interneurons and a small portion of them were motor neurons. On the other hand, our data also showed that 5-HT3AR-expressing interneurons in the BLA and PlCo included SOM or CR, but not PV. These findings were consistent with those from the 5-HT3AR expression analysis in the neocortical GABAergic interneurons^41^. However, as for the 5-HT3AR-co-positive ratio of SOM to CR, the CR expression was more than SOM expression in the neocortex^41^, while the expression of SOM was more than that of CR in the amygdala. We believe that understanding these characteristic features of 5-HT3AR-expressing GABAergic interneurons from one region to another region can help in the elucidation of the various roles of 5-HT3AR-expressing interneurons in each region.

In the present study, we found many unreported 5-HT3AR-expressing regions, such as the claustrum, efferent vestibular nucleus, and IS. These findings provide clues regarding the novel functions of 5-HT3R. To cite an example from the oral areas, 5-HT3R was reported to be involved in tooth-pulp-evoked Sp5 neuronal excitation^82^,^83^ and gustatory nerve activation^83^. In fact, in our study, strong 5-HT3AR expression was observed in the Sp5ODM (the former) and NTS (the latter). Interestingly, there were many nuclei related to the innervation of oral areas in which 5-HT3ARs were expressed: the ambitious nuclei (motor branch) and NTS (sensory branch) of the glossopharyngeal and vagus nerves, the IS and DMV (parasympathetic branch) of the glossopharyngeal and vagus nerves, hypoglossal nucleus, and facial nucleus. These findings suggest that 5-HT3R might play a significant role in intraoral serotonergic innervation. In the main salivary glands, 5-HT3AR was expressed in the IS, which innervates the parotid glands; whereas no expression was found in the medullary reticular nucleus, which innervates the sublingual and submandibular glands. The expression of 5-HT3AR was observed in many regions associated with oral innervations, but its localization was confined to specific areas.

Taken together, we have successfully not only reconfirmed previous findings but also newly identified some 5-HT3R-expressing regions through precise observational analysis using 5-HT3AR-GFP TG mice. Our anatomical evidence is of immense value in developing a complete understanding of the functions of 5-HT3AR in the serotonergic system and to understand possible therapeutic targets for CNS disorders. We believe that building a mouse brain map based on 5-HT3R expression would be helpful for future research.

## Methods

### Animals

Twelve-week-old 5-HT3AR-GFP TG mice purchased from the Mutant Mouse Regional Resource Center were used in all the experiments. They were housed at a controlled temperature (23–25 °C) and were fed standard rodent pellets and water ad libitum. Every experimental procedure was approved by the animal ethics committee of the Osaka University according to the National Institute of Health Guide for Care and Use of Laboratory Animals. Complete effort was made to minimize the number of experimental animals and to optimize their living conditions.

### *In situ* hybridization

The cDNA fragments of mouse 5-HT3AR and enhanced GFP were amplified by reverse transcriptase polymerase chain reaction (RT-PCR), and used as templates for probe synthesis. The sequences of each oligonucleotide primer are described below:

5HT3AR forward 5′-AGT TTG TGG ACG TGG GGA AG-3′

5HT3AR reverse 5′-CCA GGC TAT TCT GTC TAG GAC-3′

GFP forward 5′-ATG GTG AGC AAG GGC GAG GA-3′

GFP reverse 5′-CTT GTA CAG CTC GTC CAT GC-3′

Digoxigenin (DIG)-labelled probes were synthesized as described previously^84^ and stored at −80 °C.

The brains were removed from deep-anesthetized 5-HT3AR-GFP TG mice and immediately frozen on dry ice. The brains were sliced into 14-μm-thick sections using a cryostat microtome (HM550; Thermo Fisher Scientific, Waltham, MA, USA). Mirror image coronal sections, as well as serial coronal sections, containing the hippocampal formation were mounted on APS-coated Superfrost-Plus slides (Matsunami, Osaka, Japan) and stored at −80 °C.

After fixation with 4% paraformaldehyde (PFA) in 0.1 M phosphate buffer (PB; pH 7.4), the slides were treated with 0.1% activated diethyl pyrocarbonate for RNase inactivation, and equilibrated with 5 × Standard Saline Citrate buffer (SSC; 0.075 M sodium citrate and 0.75 M sodium chloride). The samples were pre-treated with hybridization buffer (50% formamide, 5 × SSC, 40 μg/mL salmon testis DNA) at 58 °C for 2 hours, followed by hybridization in hybridization buffer containing DIG-labelled RNA probes at 58 °C for 2 days. The slides were washed at 65 °C for 1 hour in 2X SSC and then in 0.1X SSC (SSC; 0.03 M sodium citrate and 0.3 M sodium chloride). Subsequently, the slides were incubated at 22 ± 2 °C for 2 hours with alkaline phosphatase-conjugated polyclonal sheep anti-DIG antibodies (1:5000; Roche Applied Science, Indianapolis, IN, USA) in 100 mM Tris-HCl buffer (pH 7.5) containing 0.15 M NaCl and 0.5% Blocking reagent (Roche Applied Science). After washing in 100 mM Tris-HCl buffer (pH 7.5) containing 0.15 M NaCl, the sections were treated with 0.15 M NaCl, 0.05 M MgCl_2_, 3.5 μl/ml 5-bromo-4-chloro-3-indolyl-phosphate (Roche Applied Science), and 4.5 μl/ml nitro blue tetrazolium (Roche Applied Science) in 100 mM Tris-HCl buffer (pH 9.5) for the colorimetric reaction. The colorimetric reaction was stopped using TE solution (10 mM Tris-EDTA, 1 mM EDTA; ph, 8.0). The sections were treated with 95% ethanol to prevent non-specific staining. After dehydration in an ethanol gradient (50%, 70%, 95%, and 100%), the cover slips were mounted with Entellan mounting medium (Merck KGaA, Darmstadt, Germany). All the samples were analysed using an Olympus microscope (BX53; Olympus Corporation, Tokyo, Japan).

For the mirror image sections, false-coloured images (GFP: green and 5-HT3AR: red; 5-HT3AR in the control test: green and red) and overlays of the images were prepared using the Photoshop software (Adobe Systems Incorporate, San Jose, CA, USA). The number of probe-positive and co-positive cells in the dentate gyrus (DG) region of the hippocampus was counted. The averages of the number of positive cells and the rate of co-positive cells were calculated and presented graphically. This was performed for 10 pairs of hippocampi randomly selected from three mice.

### Antibodies

The primary antibodies used for immunostaining were as follows: rabbit anti-GFP polyclonal antibody (1:1000; catalogue no. A-11122, Thermo Fisher Scientific), mouse anti-NeuN monoclonal antibody (1:200; catalogue no. MAB377, Merck Millipore, Billerica, MA, USA), rabbit anti-GABA polyclonal antibody (1:1000; catalogue no. A-2052, Sigma-Aldrich, St. Louis, MO, USA), mouse anti-parvalbumin monoclonal antibody (1:500; catalogue no. P3088, Sigma-Aldrich), rat anti-somatostatin monoclonal antibody (1:100; catalogue no. MAB354, Merck Millipore), rabbit anti-calretinin polyclonal antibody (1:500; catalogue no. A-2052, Merck Millipore), mouse anti-Glial fibrillary acidic protein (GFAP) polyclonal antibody (1:100; catalog no. g3893, Sigma-Aldrich, St. Louis, MO, USA), mouse anti-adenomatous polyposis coli (APC) monoclonal antibody (1:500; catalog no. OP80, Merck KGaA), and rat anti-CD11b (alias Integrin αM chain) monoclonal antibody (1:500; catalog no. MCA711, Bio-rad, Hercules, CA, USA).

The secondary antibodies (1:500, Thermo Fisher Scientific) used for immunostaining were as follows: donkey anti-rabbit immunoglobulin G (IgG) polyclonal antibody conjugated Alexa Fluor 488 (catalog no. A-21206) and Alexa Fluor 568 (catalog no. A-10042), goat anti-chicken IgY polyclonal antibody conjugated Alexa Fluor 488 (catalogue no. A-11039), donkey anti-mouse IgG polyclonal antibody conjugated Alexa Fluor 568 (catalogue no. A-10037), and goat anti-rat IgG polyclonal antibody conjugated Alexa Fluor 568 (catalogue no. A-11077).

### Immunofluorescence staining

The 5-HT3AR-GFP TG mice were anesthetized and transcardially perfused using 4% PFA in 0.1 M PB (pH 7.4). The dissected brains were fixed in the above fixing solution at 4 °C overnight, followed by immersion in cryoprotective solution (30% sucrose in 0.1 M PB) at 4 °C, and then frozen in dry ice. The brain samples were cut into 30-μm-thick sections, floated in 0.01 M phosphate-buffered saline (PBS), and then maintained at 4 °C until further use.

The free-floating sections were washed in 0.01 M PBS and incubated at 22 ± 2 °C for 1 hour with 0.01 M PBS containing 0.3% Triton-X and 3% bovine serum albumin to increase permeability to antibodies and inhibit non-specific staining. Subsequently, the sections were treated with GFP-specific antibodies alone or in combination with each marker antibody in the blocking buffer at 4 °C overnight. After washing thoroughly, the sections were incubated with the appropriate secondary fluorescent antibodies in 0.01 M PBS at 22 ± 2 °C for 1 hour. The sections were washed several times in 0.01 M PBS and then mounted on slides using PermaFluor (Thermo Fisher Scientific). The stained samples were analysed with a BX53 microscope and a confocal microscope (BX61 type FV1000D, Olympus Corporation). Some GFP-stained sections were counterstained with 1 μg/μL 4′,6-diamidino-2-phenylindole (DAPI; Dojindo laboratory, Kumamoto, Japan) solution before mounting. The rates of GFP-co-positive staining for NeuN, GFAP, CD11b, APC, GABA, PV, SOM, and CR were calculated using over 13 visual fields from three mice. The signals of GFP overlapped with those of NeuN and GABA almost completely, and were not those of GFAP, CD11b, APC, and PV.

### Evaluation of immunofluorescence signals

A series of coronal sections from the olfactory bulb to the sacral spinal cord were prepared for immunofluorescence staining. The staining preparations of four 5-HT3AR-GFP TG mice (n = 4) were investigated fluoroscopically and then reexamined by obtaining pictures through a low-power objective (4x magnification) under the fluorescent microscope. The expression sites were identified based on two brain atlases, the Mouse Brain in Stereotaxic Coordinates (2^nd^ edition, Academic Press Elsevier, Amsterdam, the Netherlands) and the Atlas of the Spinal Cord: Mouse, Rat, Rhesus, Marmoset, and Human (Academic Press). Observations were categorized depending on both fluorescence intensity and the number of positive cells, and were quantified (coloured) as described below: no detected signal, negative (−: white); low number of GFP-positive cells, weak (+: blue); high number of GFP-positive cells, moderately high (++: yellow); strong signal intensities in the nerve fiver as well as the soma, strong (+++: red).

## Additional Information

**How to cite this article**: Koyama, Y. *et al*. Building a 5-HT3A Receptor Expression Map in the Mouse Brain. *Sci. Rep.* **7**, 42884; doi: 10.1038/srep42884 (2017).

**Publisher's note:** Springer Nature remains neutral with regard to jurisdictional claims in published maps and institutional affiliations.

## Supplementary Material

Supplementary Table 1

Supplementary Information
