# Supplementary material for: Building a 5-HT3A Receptor Expression Map in the Mouse Brain
Source: Sci Rep. 2017 Mar 9;7:42884. doi: 10.1038/srep42884 (PMC5343592; doi:10.1038/srep42884)
Supplement: Supplementary Information [file srep42884-s2.pdf]

Supplementary Information

Title

Building a 5-HT3A Receptor Expression Map in the Mouse Brain

Authors

Yoshihisa Koyama[1], Makoto Kondo[1]*, and Shoichi Shimada[1]

Correspondence to: Makoto Kondo

e-mail: mkondo@anat1.med.osaka-u.ac.jp

A list of Supplementary Information

Supplementary Figures 1-3 and Figure Legends 1-3

Supplementary Figure 1

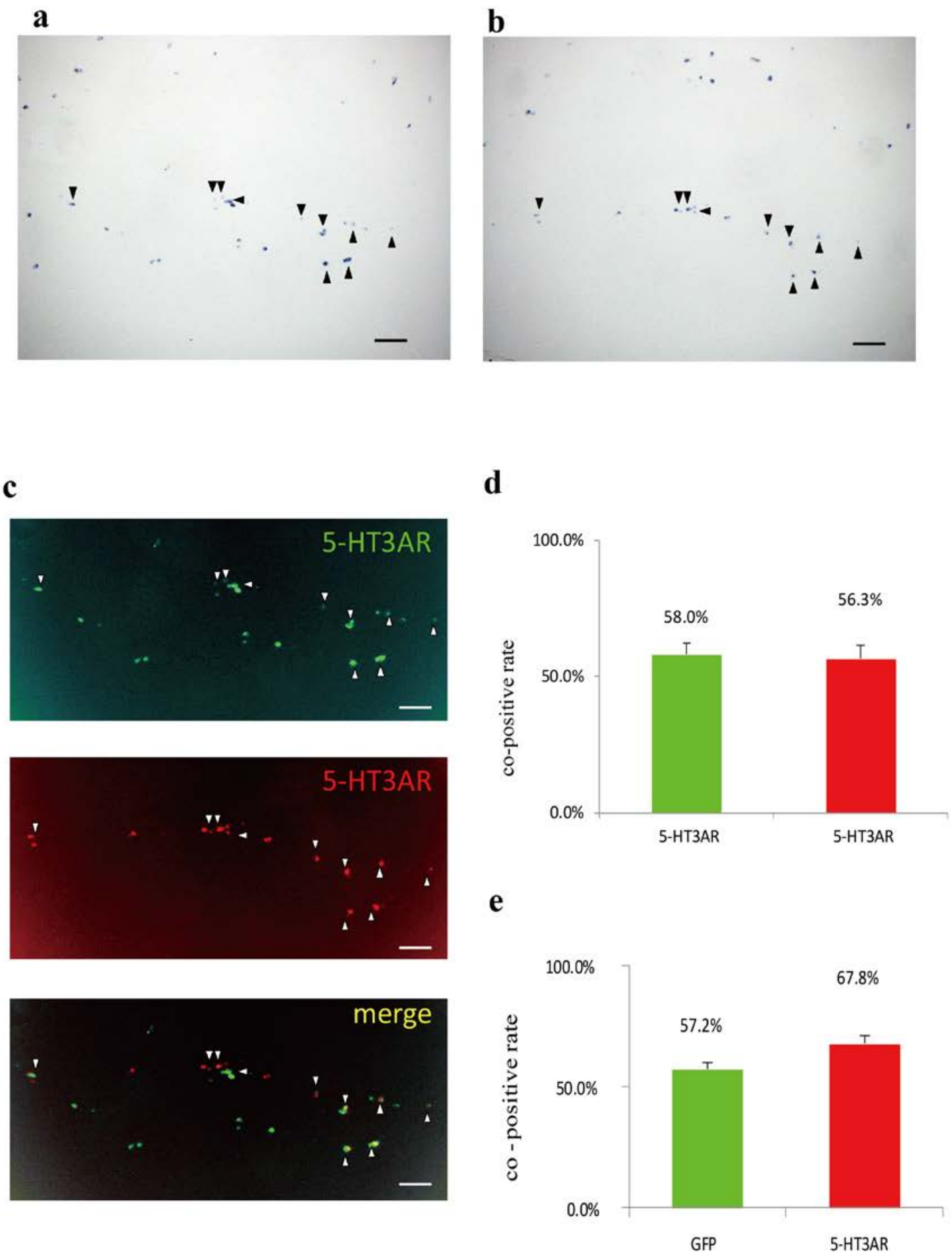

Supplementary Figure 1. Control experiments for in situ hybridization using mirror sections

(a-e) in situ hybridization analysis using mirror image sections; micrograph of 5-HT3AR mRNA (a and b); arrowheads indicate each co-positive cell; scale bar: 50 μm. (c) False colour images of in situ hybridization for 5-HT3AR mRNA (upper: green), same mRNA (middle: red), and merge (bottom); arrowheads indicate each co-positive cell; scale bar: 50 μm. (d, e) Histograms of the number of co-positive rate using same 5-HT3AR (d) and a combination of 5-HT3AR and GFP (e).

Supplementary Figure 2

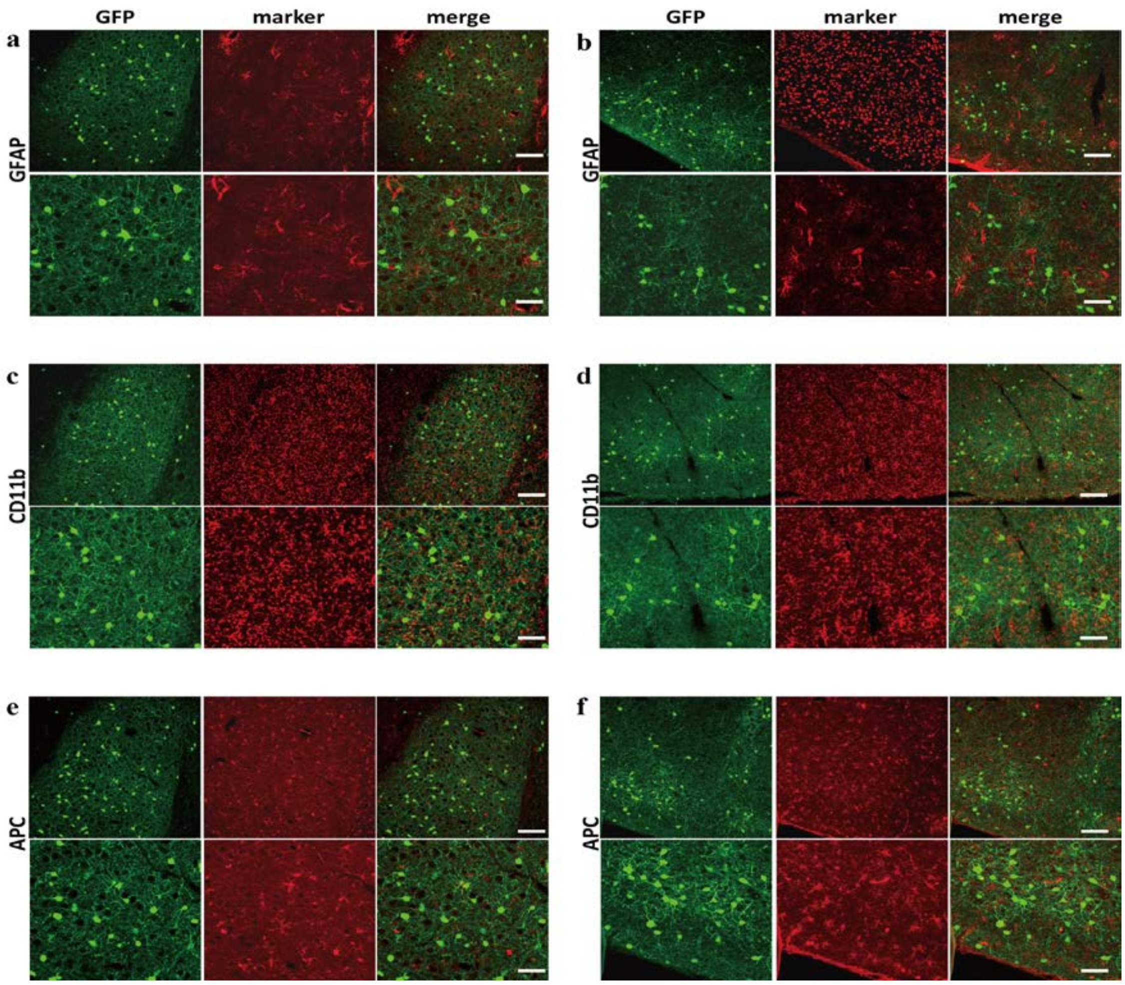

Supplementary Figure 2. Identification of the 5-HT3AR-expressing cell types

The micrographs of double staining for the basolateral amygdaloid nucleus (a, c, e), posterior cortical amygdaloid nucleus (b, d, f) of 5-HT3AR-GFP TG mice using anti-GFAP antibody (a, b), anti-CD11b antibody (c, d), and anti-APC antibody (e, f). Magnification: low (upper) and high (lower). Fluorescent signals: left (GFP), middle (each marker), and right (merge). Scale bar: 100 µm (upper) and 200 µm (bottom).

Supplementary Figure 3

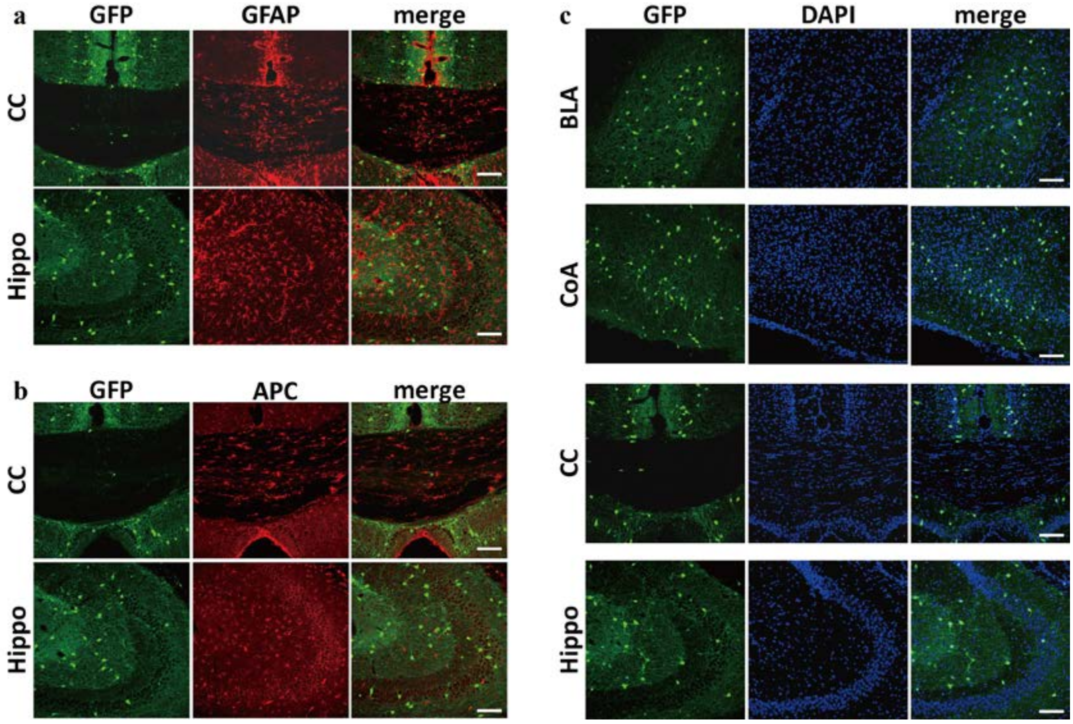

Supplementary Figure 3. Additional data for double immunofluorescent staining

(a, b) Micrographs of double staining for the corpus callosum (upper) and CA3 region of the hippocampus (bottom) in 5-HT3AR-GFP TG mice using anti-GFAP (a) and anti-APC (b) antibodies; fluorescent signals: left (GFP), middle (each marker), right (merge); scale bar: 100 µm. (c) Micrographs of counterstaining with DAPI against GFP-immunofluorescent staining for the basolateral amygdaloid nucleus (upper), posterior cortical amygdaloid nucleus (second step), corpus callosum (third step), CA3 region of the hippocampus (bottom) in 5-HT3AR-GFP TG mice; fluorescent signals: left (GFP), middle (DAPI), right (merge); scale bar: 100 µm.
